# Single-Nucleotide RNA Maps for the Two Major Nosocomial Pathogens *Enterococcus faecalis* and *Enterococcus faecium*

**DOI:** 10.3389/fcimb.2020.600325

**Published:** 2020-11-25

**Authors:** Charlotte Michaux, Elisabeth E. Hansen, Laura Jenniches, Milan Gerovac, Lars Barquist, Jörg Vogel

**Affiliations:** ^1^ Institute for Molecular Infection Biology, University of Würzburg, Würzburg, Germany; ^2^ Helmholtz Institute for RNA-based Infection Research (HIRI), Helmholtz Center for Infection Research (HZI), Würzburg, Germany; ^3^ Faculty of Medicine, University of Würzburg, Würzburg, Germany

**Keywords:** transcription start sites, RNA-seq, sRNA atlas, Gram-positive bacteria, post-transcriptional regulation

## Abstract

*Enterococcus faecalis* and *faecium* are two major representative clinical strains of the Enterococcus genus and are sadly notorious to be part of the top agents responsible for nosocomial infections. Despite their critical implication in worldwide public healthcare, essential and available resources such as deep transcriptome annotations remain poor, which also limits our understanding of post-transcriptional control small regulatory RNA (sRNA) functions in these bacteria. Here, using the dRNA-seq technique in combination with ANNOgesic analysis, we successfully mapped and annotated transcription start sites (TSS) of both *E. faecalis V583* and *E. faecium AUS0004* at single nucleotide resolution. Analyzing bacteria in late exponential phase, we capture ~40% (*E. faecalis*) and 43% (*E. faecium*) of the annotated protein-coding genes, determine 5′ and 3′ UTR (untranslated region) length, and detect instances of leaderless mRNAs. The transcriptome maps revealed sRNA candidates in both bacteria, some found in previous studies and new ones. Expression of candidate sRNAs is being confirmed under biologically relevant environmental conditions. This comprehensive global TSS mapping atlas provides a valuable resource for RNA biology and gene expression analysis in the Enterococci. It can be accessed online at www.helmholtz-hiri.de/en/datasets/enterococcus through an instance of the genomic viewer JBrowse.

## Introduction

Of the ~1,000 bacterial species that live in and on the human body, the enterococci are a group of bacteria that have become leading multidrug resistant, hospital-adapted pathogens (Lebreton et al., 2017; Jernigan et al., 2020). The enterococci comprise a genus of 49 low-GC content gram-positive commensal species within the phylum of Firmicutes that are known to occupy diverse habitats, notably in nearly every gastrointestinal core microbiota (Schloissnig et al., 2013; Lebreton et al., 2017). These bacteria are characterized not only by their ability to resist harsh conditions (extreme pH, ionizing radiation, osmotic, oxidative stress, dramatic temperature changes, etc.), they also have a “Janus face” behavior in that they can turn from a commensal into a causative agent of invasive infections.

Of particular clinical relevance are two rogue enterococcal species, *Enterococcus faecalis* and *Enterococcus faecium* (Van Tyne and Gilmore, 2014). These two distantly related enterococci have been shown to transmit a variety of antibiotic resistances to other gram-positive as well as to gram-negative bacteria *via* mobile genetic elements (Courvalin, 1994), for example, transmitting vancomycin resistance to methicillin-resistant *Staphylococcus aureus* (Weigel et al., 2003). A better understanding of how these species cause disease and spread antibiotic resistance requires a knowledge of how its genes are controlled, on both the DNA and the RNA level.

Over the years, a number of transcriptomic studies have been performed in these species. The pioneering studies used microarrays to examine expression of annotated ORFs upon culture of *E. faecalis* under diverse conditions (Aakra et al., 2005; Solheim et al., 2007; Makhzami et al., 2008; Vebø et al., 2009; Vebø et al., 2010; Abrantes et al., 2011; Mehmeti et al., 2011; Vesić and Kristich, 2013). As seen with many other bacteria, RNA-seq technology has now become the method of choice for transcriptome analysis in the enterococcus. Only one study has previously painted a global map of the *E. faecalis* transcriptome through a modified RNA-seq method (Innocenti et al., 2015). More recently, still in *E. faecalis*, transcriptomic profiling obtained after Tn-seq and RNA-seq during phage infection has revealed novel transcriptional regulator and metabolic genes essential to this process (Chatterjee et al., 2020). RNA-seq has been applied to *E. faecium* as well. RNA-seq combined with Tn-seq analysis identified genes involved in growth in human serum (Zhang et al., 2017); another RNA-seq study identified genes implicated in biofilm formation (Lim et al., 2017); and a third one used RNA-seq to describe expression changes contributing to increased pathogenicity in response to sub-inhibitory concentrations of ciprofloxacin (Sinel et al., 2017b).

One key advantage of RNA-seq over standard microarray analysis is its providing a high-resolution view of an organism’s transcriptome, which helps to define transcript borders and illuminate expression of non-annotated features such as small noncoding RNAs (sRNAs). Indeed, complementing the discovery of several transcriptional regulators of stress response and virulence genes in *E. faecalis* and *E. faecium* (Verneuil et al., 2004; Michaux et al., 2011; Lebreton et al., 2012; Top et al., 2013) interest in post-transcriptional control mechanisms in these species has been growing as well. Discovery of sRNAs is deemed particularly exciting for the Enterococci seem to lack homologs of CsrA, Hfq, and ProQ, i.e., the three major RNA-binding proteins (RBPs) associated with sRNA activity in many other bacteria (Olejniczak and Storz, 2017; Holmqvist and Vogel, 2018; Babitzke et al., 2019; Hör et al., 2020b).

Several studies have identified sRNA candidates in *E. faecalis* and *E. faecium*. Following some kingdom-wide bioinformatics-based predictions that also included 17 putative sRNAs in *E. faecalis* (Livny et al., 2008), additional candidate sRNAs were detected by microarray, cDNA amplification or northern blot analyses (Fouquier d’Hérouel et al., 2011; Shioya et al., 2011; Innocenti et al., 2015). Phenotypes of some of these sRNAs were addressed with deletion and overexpression strains, demonstrating potential roles in regulating the expression of cellular proteins in *E. faecalis* stress responses (Michaux et al., 2014). With respect to *E. faecium*, a recent study utilized RNA-seq data to predict 61 sRNAs involved in daptomycin resistance in this species, and validated ten of these by northern blot analysis (Sinel et al., 2017a).

Taken together, different techniques have been used to successfully analyze gene expression and predict sRNAs in either *E. faecalis* or *E. faecium.* To better understand and compare post-transcriptional control between these two important clinical species, however, we deemed it desirable to produce global RNA maps and sRNA annotations by means of the same RNA-seq methodology. To this end, we have here used differential RNA-seq method (dRNA-seq) (Sharma et al., 2010), which has been shown to provide single-nucleotide resolution maps of primary transcriptomes by discriminating between primary and processed 5′ RNA ends in dozens of prokaryotes (Sharma and Vogel, 2014; Hör et al., 2018). It is interesting to note here that the basic principle dRNA-seq is based on, that is differential production of cDNA after removal of the 3′ triphosphate, was first demonstrated in mapping transcription start sites in the *E. faecalis* pCF10 plasmid nearly 25 years ago (Bensing et al., 1996). Based on genome-wide annotation of transcriptional start sites (TSSs) at a single-nucleotide resolution, we define untranslated regions (UTRs) for most mRNAs genes, provide consensus sequences for sigma factor binding, and predict new sRNAs in both *E. faecalis* and *E. faecium.* Furthermore, we have validated on northern blots the expression of several of the previous and new sRNA candidates in different conditions of growth or stress related to the harsh environmental conditions that Enterococci are known to withstand. Our data should provide a useful resource for the continued exploration of transcriptional and post-transcriptional regulatory networks in two major agents of nosocomial infection. It is freely available online through an interactive instance of the genomic viewer JBrowse (Skinner et al., 2009).

## Materials and Methods

### Bacterial Growth and Sample Collection

Bacteria were grown overnight at 37°C on M17 agar plates (Oxoid, ref. **CM0817**) supplemented with 0.5% glucose. For liquid cultures, single colonies were inoculated into a 25 mL glass tube containing 8.3 mL M17 medium supplemented with 0.5% glucose, in order to keep the final proportions of one third of media to two thirds of oxygen. Cultures were grown overnight at 37°C without agitation. For dRNA-Seq samples, overnight cultures were back-diluted 1:100 into fresh M17 supplemented with 0.5% glucose and grown at 37°C without agitation to late logarithmic/early stationary phase (optical density (OD_600_ of 2.0). For northern blot analysis, growth was examined under facultative anaerobic conditions (no agitation), aerobic conditions (with agitation) and anaerobic conditions (carried out in a Whitley A35 Anaerobic Workstation). After 1:100 back-dilution of the overnight culture into the appropriate growth condition, samples in these three conditions were collected at OD_600_ of 0.5, 2.0, and at 24 h, corresponding to mid logarithmic, late logarithmic/early stationary, and stationary growth phases, respectively. Bacteria were also subjected to the following stress conditions for northern blot analysis: oxidative stress (M17 supplemented with 0.5% glucose and 2mM H_2_O_2_), acid stress (M17 supplemented with 0.5% glucose and adjusted to pH 5.5 with lactic acid), osmotic stress (M17 supplemented with 0.5% glucose and 8% sodium chloride), and bile salts (M17 supplemented with 0.5% glucose and 0.08% bile salts). In the stress assays, bacterial overnight cultures were back-diluted 1:100 into M17 medium supplemented with 0.5% glucose and grown at 37°C without agitation until an OD_600_ of 0.5. Bacteria were then transferred to the appropriate stress condition for 30 min before sample collection. All stress conditions were performed without agitation, excluding the oxidative stress condition. All samples, for both dRNA-Seq and northern blot assays, were obtained in three separate biological replicates. Upon sample collection, all samples were fixated by addition of STOP Mix [95% (vol/vol) EtOH and 5% (vol/vol) phenol], frozen in liquid nitrogen, and stored at −80°C until RNA extraction.

### Total RNA Extraction

Frozen bacterial cultures were thawed on ice, centrifuged, and cell pellets were resuspended in a lysis solution of 600 µl of 10 mg/ml lysozyme in Tris-EDTA (TE) buffer (pH 8.0) and 60 µl of 10% (w/v) sodium dodecyl sulphate (SDS). Bacterial cells were lysed by placing the samples for 10 to 12 min at 64°C in a water bath. Total RNA was extracted from the lysates using the hot phenol method with a lysis step at 37°C for 10 min in 10 mg/ml lysozyme (Aiba et al., 1981).

### Northern Blot Analysis

DNase I-treated total RNA (5 µg) was separated on a 6% polyacrylamide gel containing 7M urea. RNA was transferred onto a Hybond-XL membrane, and hybridized overnight at 42°C with γ^32^P-ATP end-labeled oligodeoxynucleotide probes (**Supplementary Table 10**). Signals were visualized on a Typhoon FLA 7000 Phosphoimager and quantified with ImageJ (EMBL software publicly available).

### TEX Treatment, cDNA Library Construction and Sequencing

cDNA library construction and sequencing of dRNA-seq samples was performed by Vertis Biotechnology AG, Munich, Germany. First, total RNA samples were examined by capillary electrophoresis on a Shimadzu MultiNA microchip electrophoresis system to check RNA quality. Total RNA samples were then fragmented by ultrasound (4 pulses of 30 sec at 4°C) and treated with T4 Polynucleotide Kinase (NEB). Each sample was then divided into two halves and one half was subjected to Terminator exonuclease treatment (+TEX) while the other half remained untreated (-TEX). For cDNA synthesis, the +TEX and -TEX RNA samples were poly(A)-tailed using poly(A) polymerase. Next, RNA 5′ Polyphosphatase (Epicentre) was used to remove the 5′PPP structures. Subsequently, an RNA adapter was ligated to the 5′-monophosphate of the RNA. First-strand cDNA synthesis was performed using an oligo(dT)-adapter primer and the M-MLV reverse transcriptase. The resulting cDNAs were PCR-amplified to around 10 to 20 ng/µl using a high fidelity DNA polymerase. PCR cycles (12–15 rounds) were performed, with barcode sequences in the 5′ sequencing adapter. The primers for PCR amplification were designed for TruSeq sequencing according to the instructions of Illumina. The cDNAs were purified using the Agencourt AMPure XP kit (Beckman Coulter Genomics) and were analyzed by capillary electrophoresis. cDNA samples were pooled for sequencing. The library pool was fractionated in the range of 200 to 600 bp *via* a differential clean-up with the Agencourt AMPure kit, and an aliquot of the size-fractionated cDNA pool was analyzed by capillary electrophoresis. The cDNA pool was single end sequenced on an Illumina NextSeq 500 system using 1 bp × 75 bp read length.

### Read Mapping

Illumina reads in FASTQ format were trimmed using Cutadapt version 1.12 with the options –m 1 –q 20 to trim 3′ bases with a Phred quality score of less than 20. Mapping was performed with the READemption pipeline (Förstner et al., 2014) version 0.4.3 using the subcommands “create”, “align” and “coverage”. The “align” command was run with the options -c -r -q -g. As reference sequences, the chromosome and plasmids for *E. faecalis* V583 (NC_004668.1, NC_004669.1, NC_004670.1, and NC_004671.1) were downloaded from the NCBI ftp server:

ftp://ftp.ncbi.nlm.nih.gov/genomes/all/GCF/000/007/785/GCF_000007785.1_ASM778v1/. Likewise, the chromosome and plasmids for *E. faecium* AUS0004 (NC_017022.1, NC_017023.1, NC_017024.1, and NC_017032.1) were downloaded from ftp://ftp.ncbi.nlm.nih.gov/genomes/all/GCF/000/250/945/GCF_000250945.1_ASM25094v1/.

### Data Visualization and Normalization

The data were visualized using the Integrative Genomics Viewer from the Broad Institute (http://software.broadinstitute.org/software/igv/). For manual annotation of TSS, each graph was normalized using the total number of reads that could be aligned from the corresponding library. To restore the original data range and to prevent rounding of small errors to zero by genome browsers, each graph was subsequently multiplied by the factor 1,000,000 of mapped reads calculated over all libraries. All data has been deposited on GEO with accession number GSE115009.

### Genome Browser Implementation

The feature annotations curated in this manuscript were used to populate a public JBrowse instance (Buels et al., 2016). RNA-seq data tracks were derived from BigWig files obtained from aligned sequencing reads using bamCoverage from deepTools (Ramírez et al., 2016).

### Prediction of TSS, Terminators, Transcripts, UTRs, and sRNAs

TSS, transcripts, terminators, UTRs and sRNAs, were predicted using the ANNOgesic pipeline version 0.6.27 (Yu et al., 2018). For each of the features, the prediction was run with the requirement that it be found in all three replicates of the dRNA-seq samples. ANNOgesic’s default parameters were used except where specified below.

Transcription start sites were predicted from wiggle files using the “tss_ps” subcommand in the ANNOgesic pipeline version 0.6.27 (Yu et al., 2018), which integrates TSSpredator version 1.06 (http://it.informatik.uni-tuebingen.de/TSSpredator. Non-normalized wiggle files were used in ANNOgesic as TSSpredator performs its own normalization. TSS were initially detected with the default parameters of ANNOgesic, but with slightly more stringent settings for the height and processing factor parameters of TSSpredator (height set to 0.5 and processing factor set to 1.2). Using the initial output as a baseline, the TSS prediction parameters were then optimized. For TSS optimization, TSS in the first 200kb of the *E. faecalis* and *E. faecium* chromosomes were manually annotated by visualizing normalized dRNA-seq wiggle files in the Integrative Genome Browser. The manual annotation was then fed back to ANNOgesic for machine learning and determination of optimal parameters. The “tss_ps” subcommand was then rerun using the optimized parameters. For *E. faecalis*, the optimized parameters were –he 0.3 –rh 0.2 –fa 1.2 –rf 0.5 –bh 0.0 –ef 1.4 –pf 4.0. For *E. faecium*, the optimized parameters were –he 0.3 –rh 0.2 –fa 2.0 –rf 0.5 –bh 0.0 –ef 1.7 –pf 0.4. Prediction based on the optimized parameters resulted in about a 20 percent increase in predicted TSS, because a number of start sites were added manually to ANNOgesic’s initial predictions. After running the optimized TSS prediction with the less stringent parameters, the output was compared to the original more stringent output. All newly predicted TSS were manually curated throughout the genomes of both bacteria to ensure that the optimized parameters were not lacking in stringency.

Transcript detection was run with the added specifications of –cf gene CDS (to compare predicted transcripts with gene and CDS features in provided annotation files) and –modify_transcript merge_overlap extend_3end extend_5end (to merge multiple transcripts within the same gene or extend 3′ or 5′ ends based on annotation files).

Terminator prediction was run using TransTermHP (http://transterm.ccb.jhu.edu/), version 2.09 and RNAfold from the ViennaRNA package version 2.1.7 (https://www.tbi.univie.ac.at/RNA/), Lorenz et al., 2011) integrated into ANNOgesic (Kingsford et al., 2007; Lorenz et al., 2011).

The sRNA prediction was run with options –m –u –cs –sf –nf –nd –sd –d tss sec_str. Option –pn was used with a promoter motif of length 45 (detected in the 50 nucleotides upstream of the TSS) to increase the ranking of sRNAs associated with a −10 box promoter element. Folding energies were predicted with ViennaRNA version 2.1.7. sRNA predictions were required to start at a TSS.

5′UTRs were predicted using the –b5 TSS command to extract the region between predicted primary and secondary TSS and CDS. Transcripts with a 5′UTR length of less than 10 nucleotides were considered to be leaderless mRNAs (**Supplementary Table 3**). 5′UTR length was initially limited to a maximum of 300 nucleotides, with an extension of up to 5 nucleotides to search an associated CDS. Subsequently, possible longer 5′UTRs with lengths up to 500 nts were annotated by hand based on orphan TSS that were clearly associated with a downstream gene, and thus should have been characterized as a primary TSS. Association with a downstream gene was based on continuous RNA-seq read coverage or the presence of a riboswitch or other leader element in the putative 5′UTR.

3′UTRs were predicted based on the regions between the end of a CDS and the end of the predicted transcript or terminator by running Annogesic with either options –b3 transcript or –b3 terminator. Parameter –et 40 was used to allow the 3′ end of transcripts to be extended or withdrawn by 40 nucleotides when searching an associated terminator. 3′UTR length was limited to a maximum of 300 with an extension of up to 20 nucleotides to search for an associated CDS set using option –t3 20. In order to limit 3′UTRs to expressed genes for higher confidence, 3′UTRs predicted based on terminators were kept only if they were also supported by the transcript prediction. For 3′UTRs predicted by both methods, the length prediction based on the terminator was kept.

### Prediction of Leader Elements in 5′UTRs

Structured RNA elements were predicted in the whole genomes of *E. faecalis* V583 and *E. faecium* AUS0004 using Infernal 1.1.3 and the Rfam database version 14.1 (Griffiths-Jones et al., 2003). Ribosomal RNA and tRNA were filtered from the results. All predicted riboswitches, RNA thermometers, leader elements and sRNAs were manually curated by viewing the predictions in the genome browser along with the RNA-seq reads, genome annotation, TSS and sRNA annotations.

### Prediction of the mRNA Targets

Either CopraRNA or IntaRNA were used to predict sRNA targets. For CopraRNA, the sRNA homologs of the following species were used: *E. faecalis* V583 (NC_004668), *E. faecalis* OG1RF (NC_017316), *E. faecalis* str. Symbioflor 1 (NC_019770), *E. faecalis* DENG1 (NZ_CP004081), *E. faecalis* D32 (NC_018221), *M. plutonius* DAT561 (NC_016938), *M. plutonius* ATCC 35311 (NC_015516), *E. casseliflavus* EC20 (NC_020995), *T. halophilus* NBRC 12172 (NC_016052), *E. hirae* ATCC 9790 (NC_018081), *E. faecium* T110 (NZ_CP006030), *E. faecium* DO (NC_017960), *E. faecium* NRRL B-2354 (NC_020207), *E. faecium* AUS00085 (NC_021994). The mRNA targets were selected based on their FDR values, base-pair energy interaction and homology in both E. faecalis V583 and E. faecium AUS0004.

### Motif Detection in Promoters and Ribosome Binding Sites

To detect promoter motifs, we performed unbiased *de novo* motif search using MEME v4.12.0 (Bailey et al., 2015) on the 50 nucleotides upstream of all genomic positions with detected TSSs (including the TSS). The search was limited to the 40 most significant motifs with widths between 4 and 9 nts, or 15 and 25 nts, or 6 and 50 nts and a motif e-value cutoff of 0.05 (example command line arguments –nmotifs40 –minw 4 –maxw 9 –evt 0.05). In order to search for possible motifs specific to sRNAs, motif detection was also run separately on the 50 nucleotides upstream of predicted sRNAs using a range of 6 to 40 nts. For those sRNAs in which a motif was not identified by this method, the motif that was detected for the other sequences was input into FIMO (part of the MEME suite) and run against only those sRNAs missing a motif for more sensitive detection using a p-value cutoff of 0.01.

Ribosome binding site motifs were predicted for mRNAs with 5′UTRs of length 10 or greater. A *de novo* motif search was run in MEME v4.12.0 in the region directly upstream of the CDS of each of these mRNAs, excluding the translation start site. For mRNAs with 5′UTR length 30 or greater, 30 nts were selected. For 5′UTRs of length 10 to 29, the entire 5′UTR was selected. The search was limited to the 15 most significant motifs with a fixed width of 6 nts and a motif e-value cutoff of 0.05.

### Prediction of RNA Motifs

Homologs to RNA sequences were searched with hidden Markov model profiles in RNAcentral.org (Wheeler and Eddy, 2013). The Rfam structure (Kalvari et al., 2018) of matches was retrieved as a scaffold for the motif prediction in RNAcentral.org. Homolog sequences of RNAs from *E. faecium* and *faecalis* were searched in GlassGO (Lott et al., 2018)and the list of retrieved sequences was collapsed to the unique ones and aligned with hierarchical clustering in MultAlin with default parameters (Corpet, 1988). Finally, sequence (co-)variation was mapped manually on top of the RNA structure model to result the motif prediction. No Rfam annotation was available for *faecalis* RNA_104 and *faecium* RNA_67, here the secondary structure was predicted by M-fold (Zuker, 2003).

### Conservation of Predicted sRNAs

To evaluate conservation of predicted sRNAs, we used 17 completed Enterococcaceae genomes available in the ENA, including strains from the species *Enterococcus faecalis, Enterococcus faecium, Enterococcus hirae, Enterococcus casseflavus, Melissococcus plutonius*, and *Tetragenococcus halophilus*. The selected genomes are available from the ENA database under the following accession numbers: AE016830.1, FP929058.1, CP002621.1, HF558530.1, CP004081.1, CP003726.1, CP002491.1, AP012282.1, AP012200.1, CP004856.1, AP012046.1, CP003504.1, CP006030.1, CP003583.1, CP004063.1, CP006620.1, CP003351. An iterated nhmmer (Wheeler and Eddy, 2013) search was run for two iterations, with a minimum e-value cut-off of 10^−7^ for sequence inclusion in the HMM. The resulting sequence alignment was used to determine percent identity with respect to the reference sequence using esl-alipid, distributed with hmmer 3.1b1. A heatmap of the computed percent identities was generated using the R superheat package with the pretty columns setting. To construct a phylogeny of the chosen Enterococcaceae strains, a collection of broadly conserved proteins of the selected genomes were extracted and aligned with phylosift version 1.0.1 (Darling et al., 2014), and a tree was built with FastTree version 2.1.10 (Price et al., 2010) using default parameters.

### Gene Functional Annotation and COG Classification

Gene functional annotations and assignment of clusters of orthologous groups (COG) terms were assigned for *E. faecalis* V583 and *E. faecium* AUS0004 using the eggNOG-mapper tool version 4.5.1 with default settings (Huerta-Cepas et al., 2016).

## Results

### Genome-Wide TSS Map

**Figure 1:**
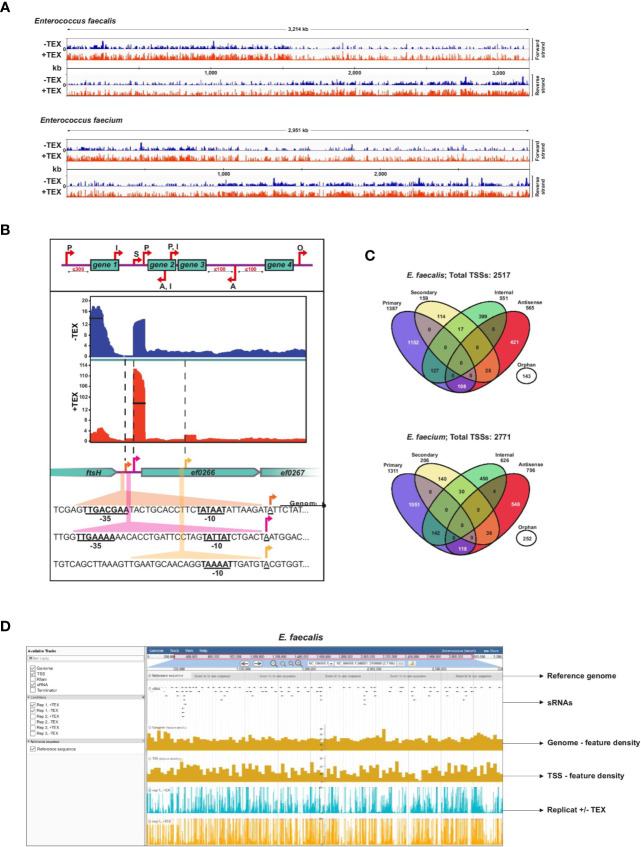
*Enterococcus faecalis* and *E. faecium* TSS revealed by dRNA-seq. **(A)**, Combined cDNA reads of a single representative experiment of three biological replicates without (blue, (−) libraries) or with (red, (+) libraries) terminator exonuclease treatment (TEX) mapped to and plotted as log_2_ values over the *E. faecalis* (top) or *E. faecium* (bottom) chromosome. All libraries were adjusted to the same scale. **(B)**, Top: representation of categories for TSS based on expression strength and genomic context: primary (P), secondary (S), internal (I), antisense (A), or orphan (O). Bottom: cDNA libraries mapped onto the cell division gene *ftsH*, a gene encoding a putative chaperonin (*ef0266*), and a gene encoding a putative zinc-binding protein (*ef0267*) with examples of detected primary, secondary, and internal TSS (indicated by arrows). σ^70^ promoter motifs positioned at −10 and −35 upstream of TSS are underlined. **(C)**, Venn diagrams showing total TSS (for chromosome and plasmids combined) detected in the *E. faecalis* (top) and *E. faecium* (bottom) genomes. A portion (~11%) of TSS associate with multiple categories, yielding 2805 total associations for the 2517 TSS detected in *E. faecalis* and 3101 total associations for the 2771 TSS detected in *E. faecium* (**Supplementary Table 1**). **(D)**, Illustrative browser view of dRNA-seq data. Shown for *E. faecalis*, but equally available for *E. faecium*. The panel presents a single replicate but all three biological replicates can be viewed in the genome browser.

Here we focus on *E. faecalis* strain V583 and *E. faecium* strain AUS0004 (Paulsen et al., 2003; Lam et al., 2012). The *E. faecalis* V583 genome is 3.4-Mb, consisting of a 3.2-Mb chromosome and 3 circular plasmids. Similarly, the *E. faecium* AUS0004 genome is 3.0-Mb, with a 2.9-Mb chromosome and three circular plasmids. To define high-resolution maps of the primary transcriptome of these two species (**Figure 1A**), we performed dRNA-seq on samples collected at late logarithmic phase (OD_600_ of 2) in three biological replicates. The dRNA-seq method (Sharma et al., 2010) enables profiling of the primary transcriptome by differentiating between primary (5′-PPP) versus processed (5′-P or 5′-OH) transcripts *via* a predictable enrichment pattern of 5′-PPP ends in Terminator exonuclease treatment (TEX)-treated RNA. To detect TSS, we analyzed sequencing reads from our TEX+ and TEX- libraries using a combination of TSSpredator (Dugar et al., 2013) and manual annotation.

TSS were classified according to five categories as previously developed (Sharma et al., 2010): primary TSS (predominant transcription start site of a gene or operon), secondary TSS (alternative start site with lower expression), internal TSS (start site within a gene), antisense TSS (start site antisense to a gene ±100 nts) and orphan TSS (not associated with a gene) (**Figure 1B**). For primary TSS detected by TSSPredator, we set a maximum distance of 300 nts from the associated gene. We subsequently characterized more distant primary TSS (300–500 nts distance) manually. An example showing primary and secondary TSS with significantly different expression levels upstream of a hypothetical chaperonin in *E. faecalis* highlights the sensitivity of this approach, and the presence of a doubly-classified internal/primary TSS downstream provides an example of overlapping transcriptional organization (**Figure 1B**).

Detection of TSS by enrichment of primary transcripts in TEX-treated libraries reveals a complex primary transcriptome for both *E. faecalis* V583 and *E. faecium* AUS0004. We detected 2,517 total TSS in the *E. faecalis* chromosome and its three plasmids, and slightly more (2,771) TSS in the *E. faecium* chromosome and its three plasmids (**Figure 1C**, **Supplementary Table 1**). A breakdown of TSS in the chromosomes versus the plasmids is also presented in **Supplementary Figure 1**. A list of all TSS detected and their classifications, including multiple associations, can be found in **Supplementary Table 1**. For *E. faecalis*, we detected significantly more TSS than in a previous study on the same strain in which only 559 TSS were detected (Innocenti et al., 2015), likely owing to increased resolution afforded by the enrichment for primary transcripts and high sequencing coverage in the dRNA-seq protocol. In both *E. faecalis* and *E. faecium*, we found that primary TSS account for the majority of TSS detected (55% in *E. faecalis* and 47% in *E. faecium*). A modest ~9% to 10% of primary TSS in both strains are also internal TSS (residing in an upstream ORF), and ~8% to 9% are also antisense TSS. After primary TSS, the most prevalent TSS categories are antisense (~22–25%) and internal (~21–22%). About 6% and 9% of TSS are orphans in *E. faecalis* and *E. faecium*, respectively, revealing transcription in many intergenic regions and potential locations of sRNAs. Secondary TSS account for ~6% of TSS in *E. faecalis* and ~7% in *E. faecium*.

### Browser

To facilitate access to our results, we provide a publicly accessible JBrowse (Buels et al., 2016) instance which includes our complete feature annotations (TSS, Rfam, sRNA, terminators) for both *E. faecalis* and *E. faecium*, and visualization of our transcriptomic data from the dRNA-seq experiment (**Figure 1D**). This, available at www.helmholtz-hiri.de/en/datasets/enterococcus, provides direct access to the underlying data, and will facilitate exploration by the scientific community.

### Genome-Wide Promoter Map

To date, four transcription factors have been identified in *E. faecalis*: σ^70^ (SigA), and three extracytoplasmic factors (ECF), i.e., SigH, SigV, and SigN (a σ^54^-like factor) (Paulsen et al., 2003). Six other ECF can be inferred from homology with predicted sigma factors in *R. matallidurans* (Nies, 2004), but have not been previously studied in *E. faecalis.* A global view of promoter regions of *E. faecalis* V583 was presented in a previous study mapping TSS (Innocenti et al., 2015), revealing promoters for SigA and SigN. However, as previously mentioned, the number of TSS detected in that study was less than in the present study, so their global view may have been incomplete. By contrast, a global view of promoter regions in *E. faecium* has never been defined. In order to better define promoter regions in both species, we performed a blind search for motifs located in the regions directly upstream of TSS using MEME (Bailey et al., 2015). In addition to illuminating a global promoter map, this method also served to provide further support for our detected TSS.

**Figure 2:**
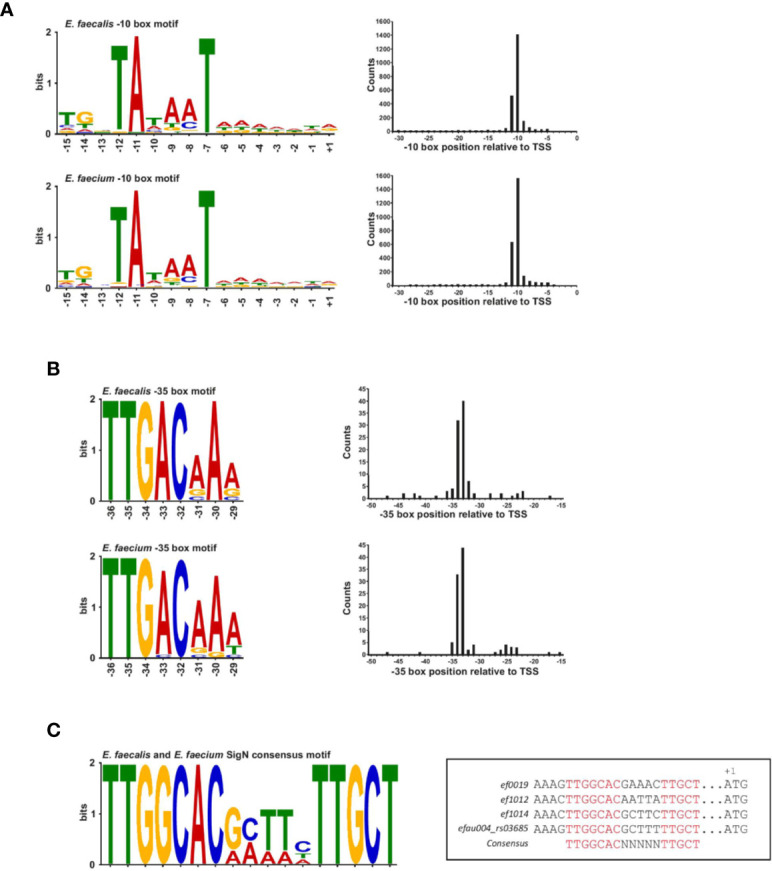
Transcription factor binding site motifs. **(A)**, The −10 box motif for the σ^70^ transcription factor detected in a blind motif search in MEME in promoter regions upstream of TSS (left). Motifs correspond to ~96% of promoter sites in *E. faecalis* (top left of the panel) and ~98% of promoters sites in *E. faecium* (bottom left of the panel). **(B)**, The −35 promoter motif for the σ^70^ transcription factor detected in a blind motif search in MEME in promoter regions upstream of TSS (left). Motifs correspond to ~4% of promoter sites in *E. faecalis* (top left of the panel) and *E. faecium* (bottom left of the panel). Positions at the right of each motifs for both panels **(A, B)** represent the median position of the motif in all promoter sites. Images were generated using Weblogo. **(C)**, Consensus SigN motif manually found in three *E. faecalis* genes and one *E. faecium* gene. The motif was generated using Meme and the specific sequences and corresponded genes locus are aligned in the box aside.

A blind motif search revealed an extended Prinbow box (tgnTAtaaT) for σ^70^ centered approximately at the −10 position for 96% of TSS in *E. faecalis* and 98% of TSS in *E. faecium* (**Figure 2A** and **Supplementary Figure 2A**). In contrast, we could detect a canonical −35 box (TTGACAAa for *E. faecalis* and TTGACaAa for *E. faecium*) for only about 4% of TSS (about 100 genes) in both strains (**Figure 2B**), suggesting that most promoters may require an accessory activator protein for activity (Hook-Barnard and Hinton, 2007). No specific motif that differed from σ^70^ found for the majority of the transcripts was identified for the sRNAs discussed further below: 148/150 of *E. faecalis* sRNAs and 125/128 *E. faecium* sRNAs were found to have a σ^70^ motif (**Supplementary Figure 2B**). Specifically, no motif was identified upstream of *E. faecalis* sRNA_037 and _102 or *E. faecium* sRNA_092, _101 and _104.

It has been observed in *E. coli* that a conserved −35 box at optimal spacing is stronger than an extended −10 box (Ponnambalam et al., 1988; Kumar et al., 1993). In line with this idea, a functional analysis of genes associated with promoters possessing conserved −35 boxes reveals genes encoding tRNAs and ribosomal RNAs, as well as genes encoding proteins involved in basic cellular functions such as metabolism, transcriptional regulation, translation, ribosomal structure and biogenesis, and cell wall formation according to the COG database (**Supplementary Table 2** and **Supplementary Figure 2C**). A few genes are also involved in cell division, replication, recombination and repair (**Supplementary Figure 2C**). Thus, while the promoters with conserved −35 boxes seem to drive basic cellular functions, they are not strictly confined to housekeeping genes, in contrast to what has been observed in other organisms (Heidrich et al., 2017). Of note, about 25% of promoters with conserved −35 boxes are associated with hypothetical proteins or genes of unknown function, pointing to potentially important as yet uncharacterized genes. Interestingly, we observed that the −10 boxes lacking a conserved −35 box are preceded by a weak AT-rich periodic sequence similar to that observed in *H. pylori* and *Campylobacter*, a feature thought to compensate for the absence of a conserved −35 motif by creating highly curved DNA (Petersen et al., 2003; Sharma et al., 2010) (**Supplementary Figures 2A, B**). It is also worth noting that the enterococcal RpoD protein lacks the so-called non-essential region (Pfam ID : PF04546), corresponding to an ~250 amino acid deletion relative to the *Escherichia coli* RpoD sequence. As this region is poorly characterized, it is unclear what (if any) effect this may have on affinity to a particular motif.

Aside from σ^70^, motifs for other transcription factors could not be identified in a blind motif search. This is not surprising given that ORFs coding for the other known sigma factors are not expressed in our growth conditions, with the exception of the ORF encoding *sigN*. We therefore searched manually for the SigN consensus sequence (−24/−12; TTGGCACNNNNNTTGCT) in promoter regions (**Figure 2C**) (Héchard et al., 2001). In *E. faecalis*, we found a perfect match to the consensus sequence in only three locations, upstream of ORFs encoding components of phosphotransferase systems (PTS), *ef0019, ef1012*, and *ef1017*, similar to what has been observed previously (Iyer and Hancock, 2012; Innocenti et al., 2015). In *E. faecium*, we found an exact match to the consensus sequence in only one location, upstream of *efau004_rs03685*, which is the first gene in a PTS operon (**Figure 2C**). Our search output a number of other imperfect, yet significant, matches to the consensus sequence, but the other ORFs all lacked an upstream SigN activator protein, which is thought to be necessary for SigN transcriptional activity (Buck et al., 2000).

### Features of 5′ and 3′UTRs and Embedded Regulatory RNA Elements

**Figure 3:**
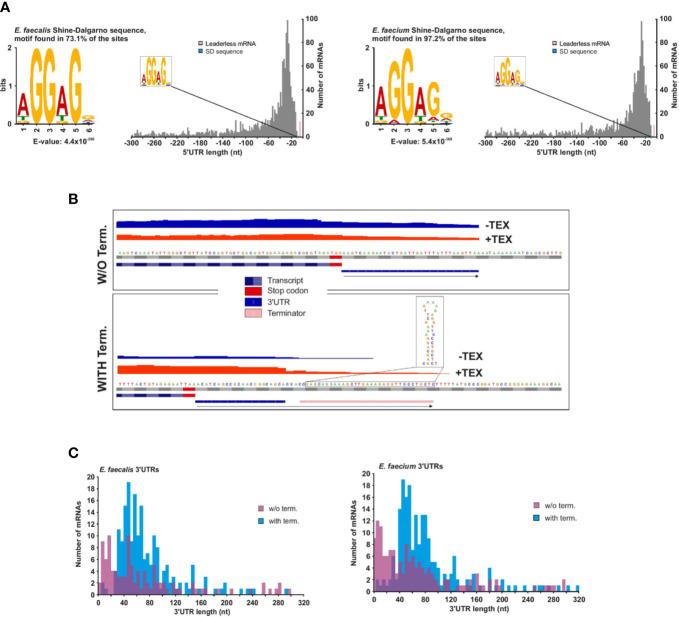
*E. faecalis* and *E. faecium* 5′ and 3′UTRs. **(A)**, Frequency of individual 5′UTR length based on 1456 and 1461 TSS (primary and secondary) of mRNAs for *E. faecalis* (left) and *E. faecium* (right), respectively. 5′UTR lengths <10 nts (pink bars) reveal 13 leaderless mRNAs in *E. faecalis* and 10 in *E. faecium* (for more details, see **supplementary Table 3**). **(B)**, Schematic indicating detection methods for 3′UTR length for mRNAs without (top) or with (bottom) a predicted Rho-independent terminator (RIT). In the absence of a predicted RIT, the length was based solely on a predicted transcript based on reads following the CDS, and a subsequent drop in coverage. In the presence of a predicted RIT, 3′UTR length was based on the predicted RIT, with the requirement that a transcript also be detected extending past the CDS. **(C)**, Frequency of individual 3′UTR length based on 1456 and 1461 TSS (primary and secondary) of mRNAs for *E. faecalis* (top) and *E. faecium* (bottom). 3′UTR lengths of zero (representing ~80% and ~77% of mRNAs with a TSS in *E. faecalis* and *E. faecium*, respectively) are not shown.

The 5′UTR (untranslated region) between the TSS and start codon influences the translational efficiency of messenger RNAs. We identified 1,456 mRNAs associated with a primary (and sometimes secondary) TSS in *E. faecalis* and 1,461 in *E. faecium* (**Supplementary Table 3**). Our TSS annotation reveals that the majority (~53%) of 5′UTRs are 14 to 42 nucleotides (nts) in length in *E. faecalis* and 12 to 40 nts in length in *E. faecium* (**Figure 3A**). In both strains, the 5′UTRs support the aGGaGg motif as the consensus Shine-Dalgarno sequence located 6 to 7 nts (median distance for *E. faecium* and *E. faecalis*, respectively) upstream of the start codon (**Supplementary Figure 3A**). Initially, we used TSSPredator’s default cutoff of 300 nts for detecting 5′UTRs, but we suspected there could be longer 5′UTRs deriving from orphan TSS that had been misannotated and were actually primary or secondary TSS. Therefore, we evaluated the distance between orphan TSS and the nearest CDS on the same strand. This revealed a number of orphans between 300 and 500 nts, then a reduction in occurrences beyond a 500 nts distance and a subsequent increase in occurrences for distances beyond 1,000 nts (**Supplementary Figure 3B**). We therefore manually checked orphan TSS lying 300 to 500 nts from a downstream CDS, and assumed that orphan TSS beyond 500 nts from a CDS are unlikely to be missed primary TSS (**Supplementary Figure 3B**). Through our manual annotation, we identified several additional 5′UTRs with length 300 to 500 nts from primary or secondary TSS that had initially been misannotated as orphans (20 in *E. faecalis*, 9 in *E. faecium*; **Supplementary Table 3**).

By examining potential correlation of 5′UTR length to cellular function, we observed, unsurprisingly, that 5′UTRs for genes known to often contain leader elements, such as those involved in translation, ribosomal structure and biogenesis (Fu et al., 2013) (COG class J) tend to be slightly longer than in other functional categories (**Supplementary Figure 3C**). In line with this observation, our Rfam search revealed L10, L20, and L21 leader elements in 5′UTRs of ribosomal proteins in both strains, an L13 leader in *E. faecalis* and an L19 leader in *E. faecium* (**Supplementary Table 4**). Additionally, in each strain, we found a single unusually long 5′UTR (686 nts in *E. faecalis* and 582 nts in *E. faecium)*, belonging to a gene encoding an amino acid ABC transporter in which the Rfam search predicted two sequential T-boxes, a known phenomenon of complex riboswitch organization (Breaker, 2008) (**Supplementary Table 4**).

Numerous riboswitches and other cis-regulatory elements have already been detected, and in some cases confirmed, in 5′UTRs of *E. faecalis* (Dar et al., 2016). However, they have not been studied in *E. faecium*, and only 6 riboswitches are included in the current annotation. We therefore took advantage of our TSS data paired with Rfam predictions to identify potential 5′UTR-based regulatory elements in all predicted 5′UTRs. First, we confirmed that we could identify validated riboswitches in *E. faecalis* with our Rfam predictions. We indeed found among our predictions the experimentally characterized SMK box riboswitch (Smith et al., 2010) and the AdoCbl riboswitch (DebRoy et al., 2014) (**Supplementary Table 4**, **Supplementary Figure 3D**). The AdoCbl riboswitch is unique in that it is encoded as part of a *trans-*acting sRNA in an intergenic region of the ethanolamine utilization locus (DebRoy et al., 2014). In addition to predicting the riboswitch, we also predicted the presence of the sRNA (**Supplementary Figure 3D**). In 5′UTRs of *E. faecium*, our Rfam predictions then revealed 13 riboswitches (including the 6 already in the annotation), 8 T-boxes, 4 ribosomal protein leader elements, and two other cis-regulatory elements (Lacto-RpoB and PyrR), all of which were expressed in our growth condition (**Supplementary Table 4**). Overall, riboswitches or leader elements are predicted in ~5% of 5′UTRs with length over 60 nts in *E. faecium*, and in ~6.5% of 5′UTRs with length over 60 nts in *E. faecalis.* The average length of 5′UTRs containing riboswitches or leader elements is 242 nts in *E. faecium* (range, 68−582 nts) and 290 nts in *E. faecalis* (range, 89−686 nts). This leaves a multitude of 5′UTRs over 60 nts as candidates for harboring potentially new riboswitches or other cis-regulatory elements.

In both strains, we found only one type of thermoregulator, the *cspA* mRNA, of which *E. faecalis* has one copy and *E. faecium* has two copies (**Supplementary Table 4**). Interestingly, while beginning at the start of the 5′UTR (which has length ~120 nts), the predicted thermoregulator extends through the coding sequence up to a Rho-independent terminator at the end of the 3′UTR (**Supplementary Figure 3D**). This prediction agrees with the finding in *E. coli* that the entire CspA mRNA acts as a thermoregulator by adopting different temperature-dependent conformations (Giuliodori et al., 2010; Zhang et al., 2018), and underscores the high evolutionary conservation of this structure (Graumann and Marahiel, 1998).

Although leaderless mRNAs are considered more common in gram-positive than gram-negative species (Vockenhuber et al., 2011), we found that less than 1% of all *E. faecalis* and *E. faecium* mRNAs have a 5′UTR less than 10 nts (13 genes in *E. faecalis* and 10 genes in *E. faecium*, **Figure 3A** and **Supplementary Table 3**). Most possess a canonical AUG start codon, but there are two examples of leaderless GUG start codons in each strain, as well as one leaderless UUG start codon in *E. faecalis.* A functional analysis did not reveal enrichment for a particular gene function for leaderless mRNAs, although there is some commonality between the strains. For example, a gene encoding a DNA polymerase III subunit are leaderless in both strains (*efau004_rs05310* and *ef2374*), as is a PTS gene (*efau004_rs10035* and *ef0457*), and several genes encoding hypothetical proteins (*efau004_rs14565*, *efau004_rs07455*, *ef3274*, *ef1448*, *ef2671*, *ef2792*, and *ef3119*) (**Supplementary Table 3**).

As with 5′UTRs, 3′UTRs can also harbor regulatory ncRNA elements. To predict 3′UTRs, we used a combination of a drop in read coverage in the dRNA-seq reads as well as predicted Rho-independent terminators (RITs, **Supplementary Table 6**). For those mRNAs with predicted RITs, we only kept those that showed some expression in our condition (**Figure 3B**). For mRNAs lacking predicted RITs, we based the prediction solely on a drop in read coverage (**Figure 3B**). Using this method, we found that ~13% and 14% of mRNAs with detected TSS in *E. faecalis* and *E. faecium*, respectively, have 3′UTRs with RITs (**Supplementary Table 5**). Of these, the majority (~65% in *E. faecalis* and ~67% in *E. faecium*) have a 3′UTR length between 40 and 90 nts (**Figure 3C**).

We detected 104 3′UTRs without RITs in *E. faecalis* (~7% of mRNAs) and 141 in *E. faecium* (~10% of mRNAs) (**Figure 3C**). The 3′UTRs without RITs were on average shorter than those with RITs, with ~75% to 76% (in *E. faecalis* and *E. faecium*, respectively) in the range of 1 to 85 nts in length likely due to the fact of only using a drop in read coverage without the possibility to extend the prediction to a terminator. In total, we detected 3′UTRs (with or without RITS) in ~20% and 23% of mRNAs with a TSS in *E. faecalis* and *E. faecium*, respectively. In two of the longer 3′UTRs with RITs in both strains, we predicted a T-box in a ~250 nts 3′UTR for a gene encoding glutamate tRNA ligase (*gltX* in *E. faecalis* and *efau004_rs13345* in *E. faecium*). Additionally, we predicted an L17 downstream element in ~180 nts 3′UTR of the 50S ribosomal protein L17 (*rplQ* in *E. faecalis, efau004_rs00122* in *E. faecium*), thought to form a feedback loop (Weinberg et al., 2010) (**Supplementary Table 4**).

### A Plethora of Small RNAs

The investigation of enterococcal sRNAs, notably in *E. faecalis*, started in the mid-1990s, around the same time that the first chromosomal sRNAs were being discovered in *E. coli*. One of the first described was the plasmid-borne *par* toxin-antitoxin system located on pAD1 (Weaver et al., 1996), homologs of which were later also identified at chromosomal loci (Weaver, 2012). The first post-genomic studies aimed at discovering sRNAs in *E. faecalis* have focused on a limited number of intergenic regions through the use of tiling microarrays or 5′tagRACE (Fouquier d’Hérouel et al., 2011; Shioya et al., 2011). To date, there has been only one global transcriptomic study in *E. faecalis* using TSS data to predict sRNAs (Innocenti et al., 2015), but no previous study employing the dRNA-seq method (Sharma et al., 2010) for mapping TSS of sRNA candidates. Moreover, out of a total of 147 predicted sRNA candidates from these transcriptomic studies in *E. faecalis*, only a few have been experimentally confirmed (Fouquier d’Hérouel et al., 2011; Shioya et al., 2011; Michaux et al., 2014). The ncRNA landscape in *E. faecium* is even less explored. One study used a global approach to search sRNAs in intergenic regions, predicting 61 sRNAs and validating 10 (Sinel et al., 2017a), but no study has used TSS data to predict sRNAs. Therefore, we set out to use our dRNA-seq data to obtain a more global validation of sRNAs in *E. faecalis*, and to predict sRNAs from TSS in *E. faecium* for the first time.

**Figure 4:**
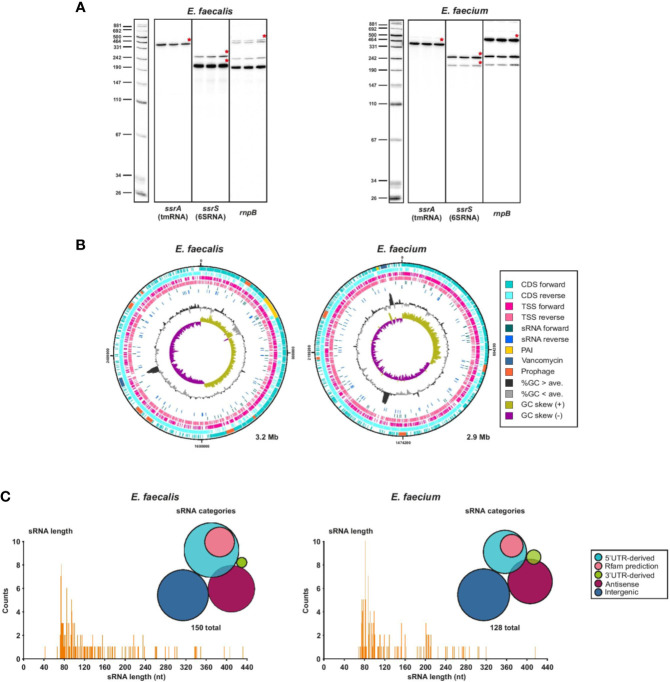
Global view of predicted sRNAs. **(A)**, Known housekeeping sRNAs were used to validate TSS samples used for dRNA-seq. Specifically, total RNA (5 µg) of 3 biological replicates of either *E. faecalis* or *E. faecium* was extracted at OD2 and analyzed by northern Blot using labeled DNA probes complementary to the mentioned housekeeping sRNAs. **(B)**, Chromosomal CDS, TSS, sRNAs, pathogenicity island (PAI) (Shankar et al., 2002; Leavis et al., 2004), vancomycin resistance region, prophages, %GC content, and GC skew for *E. faecalis* (left) and *E. faecium* (right). Features plotted over the entire chromosome with DNAPlotter. **(C)**, Frequency of the length of individual predicted sRNAs and sRNA classification categories for *E. faecalis* (left) and *E. faecium* (right). 5′UTR-derived sRNAs are derived from a primary or secondary TSS that are at the start of a 5′UTR; Rfam predictions refer to 5′UTR-derived sRNAs that are also predicted by Rfam to be either T-boxes or riboswitches. Antisense sRNAs are predicted to be antisense to another transcript.

First, to validate our RNA-seq data, we confirmed that we could detect known ncRNAs such as tmRNA, 6S RNA or RnpB which is the ribozyme element of RNase P, in our samples sent for sequencing and in the sequencing results (**Figure 4A**). As a further proof of concept, we identified non-coding transcripts (sRNA149 and sRNA155) originating from plasmids (pTEF1 and pTEF2, respectively) that appear to be homologs of the sRNA Qa, which regulates the expression of conjugation machinery on the pCF10 plasmid of *E. faecalis* (Bae et al., 2004). We also found the type I antitoxin RatA (sRNA138) (Wessner et al., 2015) expressed at a similar level to its cognate toxin mRNA encoding *txpA* (*ef3249*) (**Supplementary Table 4**). We then predicted sRNA candidates from transcripts originating from TSS in intergenic regions, antisense to annotated and non-annotated genes, and enriched in 5′ and 3′UTRs, using a cutoff of 500 nts and a maximum predicted folding energy (δG) of −0.05 kcal mol^−1^. With this method, we predicted a total of 150 ncRNAs in *E. faecalis* and 128 in *E. faecium*, mostly in the chromosomes and a few in the plasmids, and named them by number according to their order in the genome (**Supplementary Table 7**). Out of our 150 predicted sRNAs in *E. faecalis*, 83 were new, not predicted by previous studies (Fouquier d’Hérouel et al., 2011; Shioya et al., 2011; Innocenti et al., 2015). About 44% of previously predicted sRNAs did not show up in our predictions. In sum, this brings the total current list of predicted sRNAs in *E. faecalis* to about 230 sRNAs. Of our 128 predicted sRNAs in *E. faecium*, the majority (116) were new, as our predictions matched only ~20% of those previously predicted in daptomycin conditions (Sinel et al., 2017a). This brings the total current list of sRNA predictions in *E. faecium* to about 165 sRNAs.

Comparison of our sRNA predictions to the Rfam predictions added additional *in-silico* support to some of our predicted sRNAs. For *E. faecalis*, ~30% of our predicted sRNA matched an Rfam prediction. Of these, 12 corresponded to riboswitches, 6 to T-boxes, 3 to toxin-antitoxin systems, 4 to housekeeping RNAs and 19 to other sRNAs (**Supplementary Table 4**). For *E. faecium*, ~14% of our predicted sRNAs had a corresponding Rfam prediction. Of these, 3 mapped to riboswitches, 6 to T-box, 7 to sRNAs, 1 to a housekeeping RNA and 1 to another cis-regulatory element (**Supplementary Table 4**).

In both strains, we found predicted sRNAs to be evenly distributed throughout the chromosome on both strands (**Figure 4B**). The positions of sRNAs in the plasmids are less broadly distributed (**Supplementary Figure 4**). Intriguingly, we predicted several new sRNAs in the pathogenicity island (PAI) of *E. faecalis* (Shankar et al., 2002), although none are predicted within the putative PAI of *E. faecium* (Leavis et al., 2004). In *E. faecalis*, a sRNA is also predicted within the vancomycin resistance region. We checked the promoter motif found upstream of predicted sRNAs and found a conserved −10 box for σ^70^ (matching the motif found for the majority of TSS throughout the genomes) for ~96% of sRNAs in *E. faecalis* and ~91% in *E. faecium* (**Supplementary Table 7**, **Supplementary Figure 2B**). We also checked for predicted RITs and found that ~23% and ~28% of predicted sRNAs in *E. faecalis* and *E. faecium*, respectively, are predicted to end with a RIT (**Supplementary Table 7**). Most predicted sRNAs (~54% in *E. faecalis* and ~58% in *E. faecium*) are in the range of 73 to 115 nts, the longest in *E. faecalis* being 432 nts and the longest in *E. faecium* being 416 nts (**Figure 4C**).

Bacteria have developed sRNA-dependent regulatory mechanisms of mainly two kinds: sRNAs can act in *trans* where the sRNA and its mRNA target are located in different genomic regions with limited base-pairing recognition, or in *cis*, where the sRNA and the mRNA target are located in the same genomic region and complete base-pairing occurs (Waters and Storz, 2009). It has been proposed that due to the absence of a (known) global RNA chaperone such as Hfq, CsrA, or ProQ in the Enterococci, *cis*-acting sRNAs and riboswitches may be more abundant in gram-positive bacteria than *trans*-acting sRNAs, which are more common in gram-negative bacteria (Waters and Storz, 2009). Indeed, among our predicted sRNAs, less than half (~34–40%) are intergenic (*trans-*acting) (**Figure 4C**). The remaining are antisense to another transcript (~26–28%), 5′UTR-derived (~27–37%), or 3′UTR-derived (~1–3%), of which the latter two categories could be *cis* and/or *trans*-acting (Ren et al., 2017) (**Figure 4C**). However, by comparing our sRNA predictions to our Rfam predictions, we realized that ~30% of the 5′UTR-derived predicted sRNAs (including some of those that were predicted in previous studies) are actually riboswitches or T-boxes (**Supplementary Tables 7**, **8**). Nonetheless, predicted intergenic sRNAs still make up less than half of predicted sRNAs after this correction.

### Conservation Patterns of sRNAs

**Figure 5:**
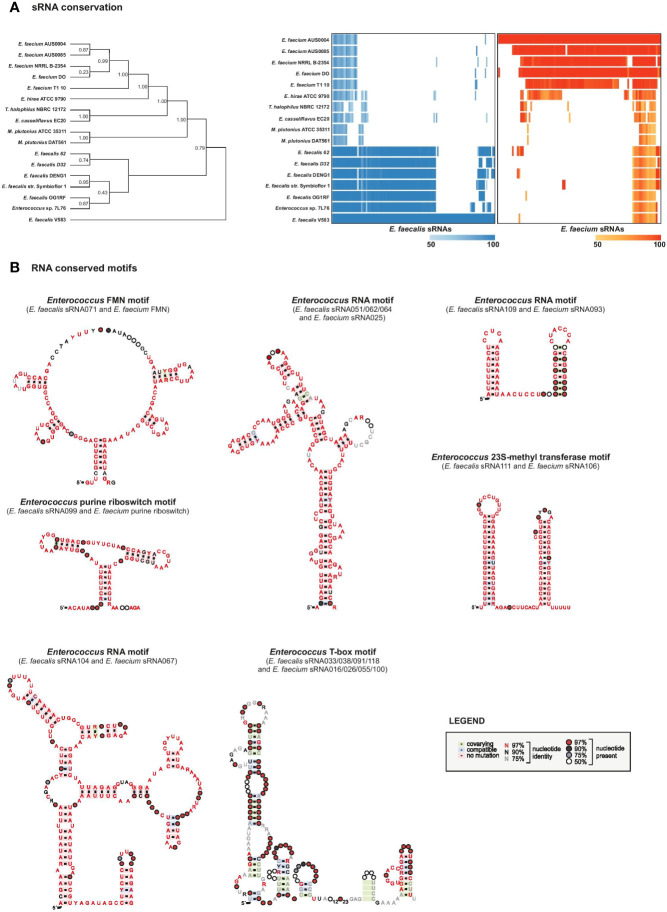
sRNAs conservation. **(A)**, sRNA conservation among 17 Enterococcaceae genomes. Phylogeny was constructed based on broadly conserved proteins in the selected genomes. The heatmap was constructed based on sequence alignments determined by an iterated nhmmer and a subsequent calculation of percent identity to the reference genome (*E. faecalis* V583, left, or *E. faecium* AUS0004, right). **(B)**, RNA motifs were generated on top of Rfam structures with RNA homologs in *E. faecalis* and *faecium* that were retrieved by GlassGO. Motifs were generated with the following number of unique sequences: purine riboswitch n=4, FMN n=34, T-box n=8, sRNA_062/_051/_064 n=25, sRNA_109/_093 n=2. For sRNA_111 and _106 (n=15) the structure was predicted with M-fold.

We evaluated conservation of our predicted sRNAs among 17 completed Enterococcaceae genomes, including strains from the species *E. faecalis, E. faecium, E. hirae, E. casseflavus, Melissococcus plutonius*, and *Tetragenococcus halophilus*. The *E. faecalis* predicted sRNAs showed high conservation in other *E. faecalis* strains, as did those of *E. faecium* with other *E. faecium* strains (**Figure 5A**, **Supplementary Table 8**). Although some sRNAs are conserved across species, we saw that conservation drops dramatically beyond the species level, in accordance with what has been observed in conservation analyses of sRNAs in other genera (Livny et al., 2008; Jose et al., 2019). Most of the predicted sRNAs that are highly conserved across all species are actually T-boxes or riboswitches, which is not surprising given that these elements are known to be highly conserved and spread though horizontal gene transfer (Grundy and Henkin, 2006).

Other sRNAs that we found to be highly conserved across *E. faecalis* and *E. faecium* species are, predictably, housekeeping RNAs including 4.5S RNA (Ffs, ~90% identity), tmRNA (SsrA, ~87% identity), 6S RNA (SsrS, ~79% identity), and 23S methyl RNA motif (*E. faecalis* sRNA_111 with *E. faecium* sRNA_106, ~90% identity) as they are known to be highly conserved (Larsen and Zwieb, 1991; Williams and Bartel, 1996; Brown and Ellis, 2005; Weinberg et al., 2007). Predicted structural motifs for these highly conserved RNAs are shown in **Figure 5B** and **Supplementary Figure 5**.

**Figure 6:**
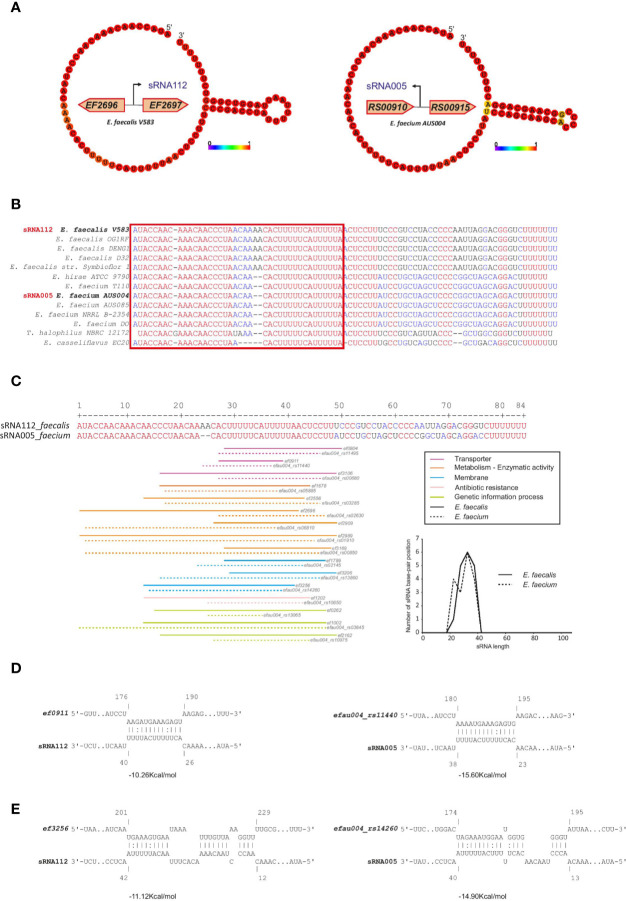
CopraRNA based target mRNA prediction for one of the most conserved sRNA couples in the Enterococcus. Potential mRNA targets of sRNA_112 (*E. faecalis*) and sRNA_005 (*E. faecium*). **(A)** Minimum Free Energy (MFE) secondary structure prediction obtained by RNAFold webserver of the two small RNAs. The nucleotides colors correspond to the base-pair probability, red being highly probable. Genomic localization of both sRNAs are display near the secondary structure predictions. **(B)** Alignment performed by MultiAlign, used as a CopraRNA input, of the two sRNAs (sRNA_112 and sRNA_005) and their homologs found in other related species. The red square highlights the putative seed region. **(C)** Distribution of the base-pairing interaction alongside the alignment of the two sRNAs. Homolog couples of the top 16 mRNA CopraRNA targets are represented and color-coded based on their Gene Ontology classification. The graphical distribution of the base-pairing location (calculated based on the middle of each interaction as a value for each interaction) has been drawn. **(D)** Interaction between an ABC transporter (*ef0911* in *E. faecalis* and *efau004_rs11440* in *E. faecium*) and the respective sRNAs. **(E)** Interaction between a lipoprotein (*ef3256* in *E. faecalis* and *efau004_RS14260* in *E. faecium*) and the respective sRNAs. The calculated energy between each interaction is written under each represented sRNA-mRNA base pairing.

In addition, we found high conservation between *E. faecalis* sRNA_109 with *E. faecium* sRNA_093 (~80% identity) with unknown function but similar to Rfam Enterococcus sRNA_084 (shown in **Figure 5B**) and *E. faecalis* sRNA_112 with *E. faecium* sRNA_005 (~81% identity, also predicted in (Sinel et al., 2017a) with unknown function. Alignment of sRNA homologs for this later couple showed the first 50 nts being highly conserved among the different species chosen for the analysis (**Figures 6A, B**). In order to identify putative targets which could help to point out a potential function, CopraRNA analysis (for Comparative prediction algorithm for small RNA targets) was undertaken (**Figure 6C**) (Wright et al., 2014). Based on a statistical model, CopraRNA compiles a target prediction list by screening targets for homologous sRNA sequences from different species; it takes into consideration the evolutionary distances between the analyzed species. Very interestingly, the interaction with putative mRNA targets where homologs were found in both *E. faecalis* V583 and *E. faecium* AUS0004 happened in the conserved regions, as illustrated with the sRNA112/sRNA005 pair (**Figure 6**). Even though assessing a function to this conserved sRNA requires further work, the pathways of the predicted mRNA targets point out a potential role in regulating membrane-associated proteins (notably lipoproteins, ABC transporters activities), metabolism (notably succinate dehydrogenase activity) or genetic information processes (protein S1) (**Figure 6C**).

Similar predictions can be made for other sRNAs. A list of the top ten conserved sRNAs in each species can be found in **Supplementary Table 8** and their top three mRNA predicted targets by IntaRNA (software which predict interactions in single species) can be found in **Supplementary Table 9**. An alignment of the 23S methyl RNA motif sRNA, which shows the highest conservation across species, and alignments of another sRNA in each of *E. faecalis* (sRNA_009) and *E. faecium* (sRNA_044), all of which we validated by northern blot, are shown in **Supplementary Figure 6**. The conservation analysis also revealed that in each species, we may have missed several sRNAs. Two sRNAs that were predicted in *E. faecalis* with hits in *E. faecium* species with ~78% to 95% identity, in similar genetic contexts and which were expressed (alignments shown in **Supplementary Figure 7**). Similarly, six sRNAs that were predicted in *E. faecium* had hits in *E. faecalis* species with ~63% to 82% identity, all expressed and in some cases in similar genetic contexts and/or with predicted terminators (alignments shown in **Supplementary Figure 7**).

### Northern Blot Validation of sRNA Expression

**Figure 7:**
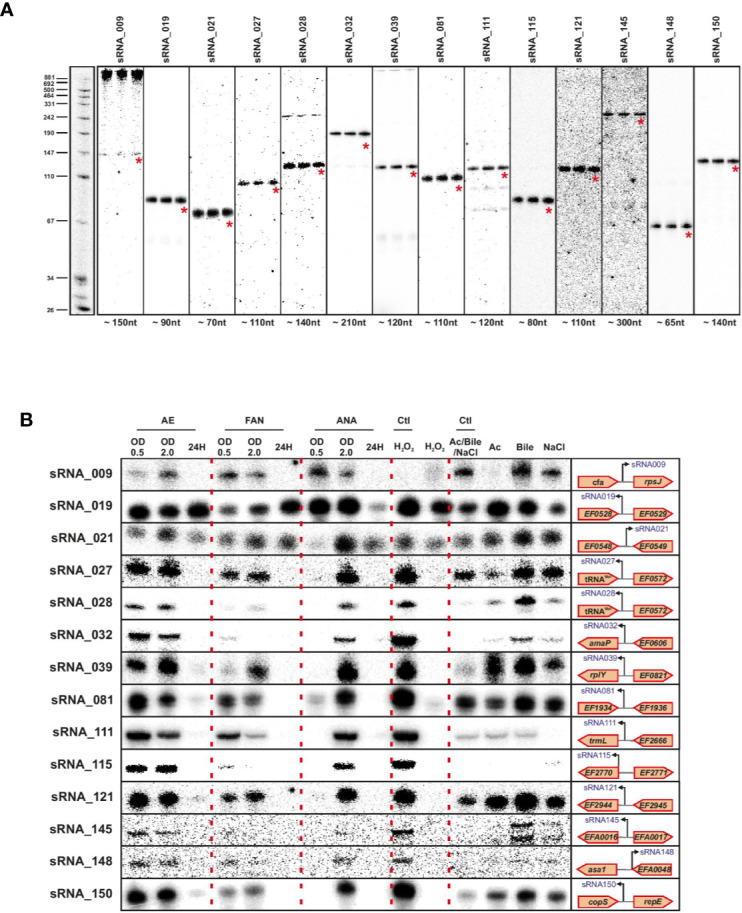
Validation of predicted sRNAs of *E. faecalis* by Northern blot. Probing of selected *E. faecalis* candidate sRNAs was performed using total RNA used for dRNA-seq and total RNA extracted during different growth phases and stress conditions. Specifically, total RNA (5 µg per lane) was extracted at OD 2 **(A)**, under different growth phases under aerobic (AE), facultative aerobic (FAN) or anaerobic (ANA) conditions or under different stress conditions (oxidative stress 2mM H_2_O_2_; acid stress pH5.5; bile stress 0.08% bile salts and osmotic stress 8% NaCl **(B)**. Labeled DNA probes complementary to the sRNAs were used to detect them.

**Figure 8:**
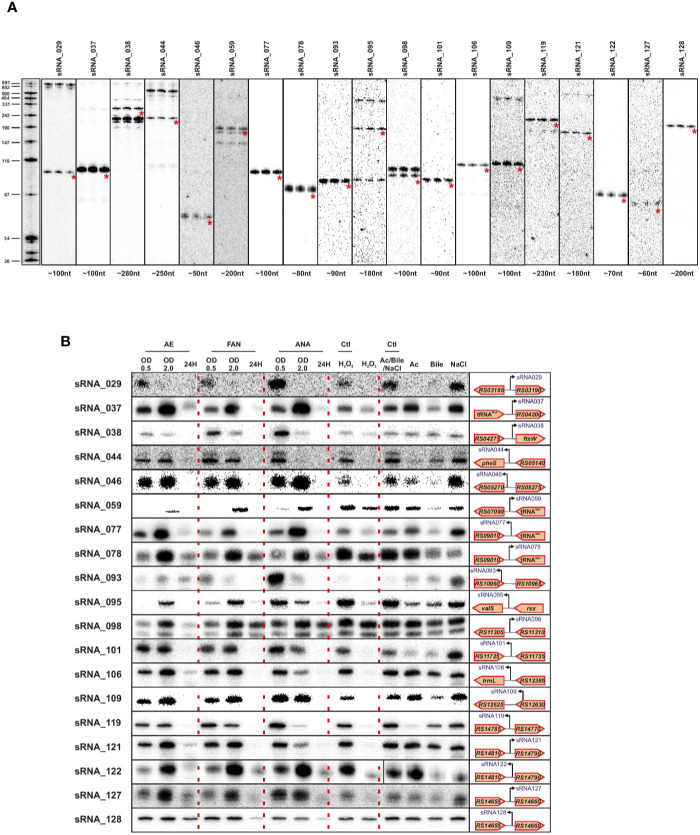
Validation of predicted sRNAs of *E. faecium* by Northern blot. Expression analysis of selected *E. faecium* candidate sRNAs was performed using total RNA used for dRNA-seq and total RNA extracted during different growth phases and stress conditions. A similar procedure as described in **Figure 7** was used. Specifically, total RNA (5 μg per lane) was extracted at OD 2 **(A)**, under different growth phases under aerobic (AE), facultative aerobic (FAN) or anaerobic (ANA) conditions or under different stress conditions (oxidative stress 2mM H_2_O_2_; acid stress pH5.5; bile stress 0.08% bile salts and osmotic stress 8% NaCl **(B)**. Labeled DNA probes complementary to the sRNAs were used to detect them.

We validated a sampling of predicted sRNAs from ANNOgesic by northern blot for *E. faecalis* and *E. faecium* in normal growth and stress conditions. We selected candidate sRNAs from ANNOgesic based on their expression and location in the genome (i.e. selecting some from intergenic, antisense, 5′UTR and 3′UTR regions). For *E. faecalis*, we tested 19 predicted sRNAs by northern blot (**Figure 7A**). We detected ~74% (14/19) by northern blot; 5 were undetectable. Of the 14 detectable sRNAs, ANNOgesic’s nts length prediction was longer for 9 sRNAs, shorter for 4 sRNAs, and a perfect match with the northern blot for 1 sRNA. For overall comparisons between ANNOgesic’s length prediction and northern blots, the predicted lengths of 10/14 sRNAs (71%) were within +/- 10% agreement, 11/14 sRNAs (79%) were within +/- 15% agreement, and 3/14 (21%) had greater than 15% discrepancy. For *E. faecium*, we tested 20 predicted sRNAs by northern blot (**Figure 8A**). We detected 95% (19/20), with only one prediction undetectable. Of the 19 detected sRNAs, ANNOgesic’s length prediction was longer for 15 sRNAs, shorter for 3 sRNAs, and a perfect match for 1 sRNA. Overall for *E. faecium*, 12/19 were within +/- 10% agreement in nts length between ANNOgesic and northern blot, 15/19 were within +/- 15% agreement, and the remaining 4/19 had greater than 15% discrepancy. mRNA target prediction using IntaRNA (Busch et al., 2008) have been done for all the validated sRNAs in both species and the top three mRNA targets can be found in **Supplementary Table 9**.

In order to have a global picture of the potential role these sRNAs could have in different cellular processes, we cultivated the two bacteria in question under conditions of stress that these commensal bacteria could encounter, such as oxygen reduction or deprivation, and oxidative, osmotic, acid or bile stresses. The results presented in **Figures 6B** and **7B** clearly show differences in expression for almost all sRNAs tested, depending on the stress applied. Even if further work will be needed to confirm the role of each sRNA in the different stress responses, the observed link between the newly validated sRNAs and a stress response—as has been observed for others sRNAs of the same species (Shioya et al., 2011; Sinel et al., 2017a) emphasizes the potential that these sRNAs have regulatory roles in stress responses in *E. faecalis* and *E. faecium*.

## Discussion

The main purpose of this work has been to provide a previously lacking global resource (TSS and noncoding RNA suite) for the comparative study of *E. faecalis* and *E. faecium*, which are two bacteria that have emerged from the astounding diversity of the core gut microbiota to become leading multi-drug resistant hospital pathogens (Van Tyne and Gilmore, 2014). The successful emergence of both of these species can be explained by genomics studies which have revealed their genomes to be over 25% larger than other commensals, notably through accretion of numerous mobile elements including prophages, insertion sequence elements, plasmids, pathogenicity islands and resistance genes such as vancomycin (Paulsen et al., 2003; Lam et al., 2012). Indeed, *E. faecalis* and *E. faecium* have been known to donate resistance to a variety of antibiotics to other gram-positive and also gram-negative bacteria, including the transmission of vancomycin resistance to *Staphylococcus aureus* (Courvalin, 1994; Weigel et al., 2003). However, while genomics have been a powerful tool, from establishing the first annotations of these bacteria to drawing their evolutionary history with the aim of understanding their phenomenal capacities to adapt to hospital environments (Lebreton et al., 2017), the next step in unveiling their complexities came with the advent of whole transcriptome RNA sequencing.

Several studies have provided transcriptional landscapes for both *E. faecalis* and *E. faecium*, but high-resolution maps of the primary transcriptome, including transcription start sites, UTRs, terminators, and sRNA predictions were still missing. Here, using TSS predator with manual annotation, we succeeded in mapping a total of 2,517 TSS in *E. faecalis* and 2,771 in *E. faecium*. While no TSS atlas has been done before in *E. faecium*, an earlier study based on a tagged sequencing method recorded 559 TSS on *E. faecalis* (Innocenti et al., 2015). This discrepancy regarding the TSS number reflects the resolution of the method used here based on the enrichment of the primary transcripts coupled with the high coverage provided by the sequencing depth.

Establishing a global atlas of sRNAs has been successfully paired with TSS mapping in other species (Sharma et al., 2010; Dugar et al., 2013; Heidrich et al., 2017). Even though some sRNAs have already been discovered – and some characterized – by other methods in both *E. faecalis* and *E. faecium* (Fouquier d’Hérouel et al., 2011; Shioya et al., 2011; Innocenti et al., 2015; Sinel et al., 2017a), our TSS data provided the opportunity to: (i) cross-check our list with previous data to confirm already discovered sRNAs and highlight new ones, (ii) have a complete and accurate list of sRNAs found in a physiological non stress-dependent condition (rich media at OD2), (iii) validate some of the new and most interesting ones.

For *E. faecalis*, 83 out of the 150 predicted sRNAs found in our dataset were new whereas the vast majority of the *E. faecium* ones have not been reported yet. This discrepancy in the results and numbers can be explained by the different methods used to identify them but also by the different culture conditions. Indeed, for *E. faecalis*, the previous studies were done under conditions that are similar to the physiological conditions we chose here (intergenic tiling array has been performed at various OD including OD2 and under different stress conditions (Shioya et al., 2011) and the 5′tag race and tagged sequencing studies have both been conducted at late exponential phase in static and under agitation (Fouquier d’Hérouel et al., 2011; Innocenti et al., 2015). It is then not surprising to find commonalities in more than half of the sRNAs found. However, for *E. faecium*, the only previous study that identified sRNAs was performed under antibiotic treatment which is certainly why, in addition to the different method used, the majority of the sRNAs we predicted were new compared to those found in the presence of daptomycin (Sinel et al., 2017a).

On a more global picture, our data suggest *E. faecalis* and *E. faecium* sRNAs share some common features with sRNAs from other species (Storz et al., 2011), in terms of their size and even distribution throughout the genome. About 98% of predicted sRNA in both species have a conserved −10 box for σ70, which does not differ from the rest of the TSS, and around 25% of sRNAs are followed by rho independent terminators.

Conservation of sRNAs is an essential feature to examine, especially in species like *E. faecalis* and *E. faecium* where very little is known about sRNA mode of action and regulation. Finding an sRNA to be highly conserved and potentially already mechanistically described in other species could lead to a better comprehension of its function. Our conservation analysis of the sRNAs among 17 Enterococcaceae species showed no conserved features with other species for most of the sRNAs, which is not surprising based on results of other conservation studies (Livny et al., 2008; Jose et al., 2019). Also unsurprisingly, the most conserved sRNAs were T-box or riboswitches which are found in very conserved regions and known to be spread through horizontal genes transfer (Grundy and Henkin, 2006). Other highly conserved sRNAs were already known and described to be highly conserved, such as tmRNA, 4.5S RNA or 6S RNA, which are not only conserved in Enterococcaceae but also among other gram-positive and negative bacteria as they are part of essential processes (Larsen and Zwieb, 1991; Williams and Bartel, 1996; Brown and Ellis, 2005). More intriguing, 3 sRNAs in particular have been found highly conserved between *E. faecalis* and *E. faecium* which could be interesting to follow up, along with numerous T-boxes and riboswitch sRNAs found in regions susceptible to be transferred and used by recipient bacteria – future studies could thereby unveil potential conserved post-transcriptional mechanisms for both species.

The conservation analysis also revealed that in each species a few sRNAs may have been missed: three sRNAs that were predicted in *E. faecalis* hid hits in *E. faecium* species, and six sRNAs that were predicted in *E. faecium* had hits in *E. faecalis* species. Possible causes of the lack of detection include the following: (i) for those with hits in 5′UTR regions, continuous read coverage to the transcript of the downstream CDS with only a minimal drop in coverage following the potentially missed sRNA could have prevented a prediction *via* the parameters used, (ii) in some cases overlap with a hypothetical protein on the same strand could have prevented detection. In several cases these regions turned out to contain predicted T-boxes. It is therefore possible that more sensitive parameters could have detected more sRNAs, however there would undoubtedly also be more false predictions.

Another important aspect of an RNAs is its location, as this can be an indicator for its potential post-transcriptional mechanism. Two major types of regulatory sRNAs have been extensively described to date: (i) sRNAs acting in *trans* with a mRNA target at a different location and (ii) sRNAs acting in *cis* with a mRNA target usually in close proximity. If in *cis* regulation, the extended base-pairing often due to the intrinsic antisense nature of the sRNA enables specific regulatory action, whereas for *trans* regulation the base-pairing is usually quite reduced and the *trans* sRNA can have multiple mRNA targets. Long base-pairing of *trans*-acting sRNAs is also not needed since for the vast majority of them, the match between the sRNA and its mRNA target is facilitated by an RBP helper (Waters and Storz, 2009). Those RBPs such as Hfq, ProQ or CsrA have been studied extensively and are known to be major actors, together with the sRNA regulators, of post-transcriptional regulation (Holmqvist and Vogel, 2018). However, while those RBPs are quite conserved in gram-negative bacteria with similar functions, notably in *Salmonella* and *E. coli*, it is not the case in gram-positive bacteria. For example, a recent Grad-seq analysis of *Streptococcus pneumoniae* found no evidence for a general sRNA-binding protein (Hör et al., 2020a). For the few RBPs that have been found in a gram-positive bacteria, their functions seem to be very different (Nielsen et al., 2010; Gerovac and Vogel, 2019; Pagliuso et al., 2019; Goodson et al., 2020; Sinha et al., 2020). Therefore, another sRNA dependent mode of regulation without protein helpers should be investigated.

Validation of the sRNA predictions demonstrated a positive signal for 14 out of 19 tested in *E. faecalis* and 19 out of 20 for *E. faecium*, showing the reliability of our approach. Regarding the precision of the approach, ANNOgesic’s length prediction and that seen by northern blots differed by no more than +/- 15% for three-quarters of the sRNAs probed. Given that our TSS data were generated in only one condition, we expect that this will not be completely comprehensive. The growth condition we chose also has some unique features, for instance the Fsr system involved in quorum sensing and biofilm formation is highly expressed in agreement with previous work in late log phase (Bourgogne et al., 2006). Knowing that *E. faecalis* and *E. faecium* can survive extremely harsh conditions and can exist in a variety of environment habitats, we therefore examined the expression of validated sRNAs under different stress conditions. In line with the discrepancy between sRNAs found under daptomycin treatment versus sRNAs found in rich media at an OD_600_ of 2 (Sinel et al., 2017a), expression changed for all the sRNAs in almost all the stresses tested implying that those sRNAs may be involved in mechanisms allowing *E. faecalis* and *E. faecium* to withstand stressful environments. This also suggested that we potentially missed some sRNAs tightly linked to a specific stress. In this regard, it is worth noting that very few of our sRNAs appeared to be expressed during starvation (24 h time point, **Figure 8B**); whether there are additional sRNAs that are activated under this condition will require further investigation.

In addition to predicting sRNAs, the TSS annotation also enabled us to better characterize 5′UTRs and 3′UTRs. Several long 5′UTRs corresponding to genes involved in translation, ribosomal structure and biogenesis were found to contain conserved leader elements. Numerous other 5′UTRs are long enough to harbor as-yet-uncovered regulatory elements. We found less than 1% of mRNAs to be leaderless, with no clear commonality among them. The detection of 3′UTRs was useful not only to provide a general idea of the landscape but also as it enabled us to localize several ncRNA elements to these regions.

In conclusion, we believe that our single nucleotide resolution TSS maps constitute a rich, accurate and precise source of information for the two opportunistic pathogens *E. faecalis* and *E. faecium*. Supported by systemic bioinformatical analysis coupled with manual curation, this atlas also constitutes a strong source of information regarding sRNAs. These data, available *via* an interactive genome browser, provide a framework and a solid basis for better further analysis of individual genes and sRNAs in these two important Gram-positive bacteria.

## Data Availability Statement

The datasets presented in this study can be found in online repositories. The names of the repository/repositories and accession number(s) can be found in the article/**Supplementary Material**.

## Author Contributions

CM, EH, LB, and JV designed research. CM, EH, and MG performed research. CM, EH, LJ, LB, and JV analyzed data. CM, EH, and JV wrote the paper with the input of all the authors. All authors contributed to the article and approved the submitted version.

## Funding

JV and LB received funding from the Bavarian BioSysNet program. This work was also supported by a Deutsche Forschungsgemeinschaft Leibniz Award to JV (DFG Vo875/19).

## Conflict of Interest

The authors declare that the research was conducted in the absence of any commercial or financial relationships that could be construed as a potential conflict of interest.

## Supplementary Material

Supplementary Figure 1
*Enterococcus faecalis* and *E. faecium* TSS classifications. Representation of categories for TSS based on expression strength and genomic context assigned by TSSPredator for the chromosome and plasmids in *E. faecalis* **(A)** and *E. faecium* **(B)**: primary (P), secondary (S), internal (I), antisense (A), or orphan (O).

Supplementary Figure 2
*E. faecalis* and *E. faecium* promoter motifs for detected TSS. **(A)** The −10 box motif for the σ^70^ transcription factor detected in a blind motif search in MEME in promoter regions upstream of TSS (left). Motifs correspond to ~96% of promoter sites in *E. faecalis* (top left of the panel) and ~98% of promoters sites in *E. faecium* (bottom left of the panel). A weak AT-rich periodic pattern is observed upstream of the −10 box (indicated by the dashed line). The −35 promoter motif for the σ^70^ transcription factor detected in a blind motif search in MEME in promoter regions upstream of TSS (right). Motifs correspond to ~4% of promoter sites in *E. faecalis* (top right of the panel) and *E. faecium* (bottom right of the panel). Positions indicated represent the median position of the motif in all promoter sites. E-values for the motifs (calculated by MEME) are indicated. **(B)** Promoter motifs for predicted sRNAs in *E. faecalis* (top) and *E. faecium* (bottom) detected by a blind motif search in MEME exhibit a conserved −10 box and a weak −35 box for the σ^70^ transcription factor. Motifs were found in ~96% and ~91% of sites for *E. faecalis* and *E. faecium* by this method respectively. Additional sRNA promoter sites matching this motif were found using FIMO, bringing the total numbers to ~98% of sRNAs for both *E. faecalis* and *E. faecium.* A weak AT-rich periodic pattern is observed upstream of the −10 box (indicated by the dashed line). Positions indicated represent the median position of the motif in all promoter sites. E-values for the motifs (calculated by MEME) are indicated. **(C)** Frequency of gene functions corresponding to promoters with conserved −35 box promoter motifs for *E. faecalis* (left) and *E. faecium* (right). Functional annotations are according to Clusters of Orthologous Groups (COGs). COG letters are defined as follows: [C] energy production and conversion; [D] cell cycle control, cell division, chromosome partitioning; [E] amino acid transport and metabolism; [F] nucleotide transport and metabolism; [G] carbohydrate transport and metabolism; [H] coenzyme transport and metabolism; [I] lipid transport and metabolism; [J] translation, ribosomal structure and biogenesis; [K] transcription; [L] replication, recombination and repair; [M] cell wall/membrane/envelope biogenesis; [O] post-translational modification, protein turnover, and chaperones; [P] inorganic ion transport and metabolism; [Q] secondary metabolites biosynthesis, transport, and catabolism; [S] function unknown; [T] signal transduction mechanisms. Genes encoding tRNA and rRNA were also found. Genes indicated as [Un] (unassigned) were not found in the COG database. Colors indicate groups of COG metacategories: metabolism (green); cellular processes and signaling (pink); information storage and processing (blue).

Supplementary Figure 3 Features of 5′UTRs. **(A)** Shine-Dalgarno (SD) sequence motif for each species found in a blind MEME search. The motif shown corresponds to ~73% of sites in *E. faecalis* and ~97% of sites in *E. faecium*, and the E-values (calculated by MEME) are indicated below. **(B)** Frequency of the distance from individual orphan TSS to the nearest downstream CDS on the same strand for all 163 orphan TSS in *E. faecalis* (red) and all 266 orphan TSS in *E. faecium* (blue). Distance frequencies are grouped in ranges, where each bar indicates a range. The minimum in each range is indicated below each bar, and the next range begins at the next bar. **(C)** 5′UTR length box and whisker plots for the Clusters of Orthologous Groups (GOG) classification for *E. faecalis* (left) and *E. faecium* (right). A COG functional class was assigned to each 5′UTR based on the downstream gene, and the 5′UTRs were subsequently grouped by function. The box plots show the UTR lengths for detected COG functional classes as well as those of unknown function and those that could not be found in the COG database. The boxes ranges from the lower to upper quartile, the line in the middle of each box indicates the median, and the plus symbol indicates the mean. Whiskers indicate the 5^th^ to 95^th^ percentile of the data, and circles indicate outliers beyond this range. COG letters are defined as follows: [C] energy production and conversion; [D] cell cycle control, cell division, chromosome partitioning; [E] amino acid transport and metabolism; [F] nucleotide transport and metabolism; [G] carbohydrate transport and metabolism; [H] coenzyme transport and metabolism; [I] lipid transport and metabolism; [J] translation, ribosomal structure and biogenesis; [K] transcription; [L] replication, recombination and repair; [M] cell wall/membrane/envelope biogenesis; [N] cell motility; [O] post-translational modification, protein turnover, and chaperones; [P] inorganic ion transport and metabolism; [Q] secondary metabolites biosynthesis, transport, and catabolism; [S] function unknown; [T] signal transduction mechanisms; [U] intracellular trafficking, secretion, and vesicular transport. Genes indicated as [Un] (unassigned) were not found in the COG database. Colors indicate groups of COG metacategories: metabolism (green); cellular processes and signaling (pink); information storage and processing (blue). **(D)** Rfam predictions for the AdoCbl riboswitch (left) and the CspA mRNA thermometer (right). Combined cDNA reads without (blue, (-) libraries) or with (red, (+) libraries) terminator exonuclease treatment are mapped to and plotted as log_2_ values on the *E. faecalis* chromosome. Within each part (left and right) libraries were adjusted to the same scale, but the maximum heights shown are different for untreated and treated libraries to facilitate viewing. Orange arrows indicate TSS. On the left, the predicted AdoCbl riboswitch is derived from the first half of a predicted intergenic sRNA (pink) with the *eutG* locus. The dotted black line indicates where the sRNA was previously found to end experimentally (DebRoy et al., 2014). The upstream gene (*ef1637*) encodes ATP cob(I)alamin adenosyltransferase; the downstream gene (*ef1635*) encodes propanol dehydrogenase PduQ. On the right, fading in the treated sample indicates that the bar continues and is cut to facilitate viewing. The *cspA* thermometer (pink) is predicted to comprise the entire CspA mRNA, including the coding sequence (green, *ef1367*), the 3′UTR and a predicted rho-independent terminator.

Supplementary Figure 4 Circular view of *E. faecalis* and *E. faecium* plasmid features. Plasmid CDS, TSS, sRNAs, vancomycin resistance region, prophages, %GC content, and GC skew for *E. faecalis* (top) and *E. faecium* (bottom). Features plotted over each entire plasmid with DNAPlotter.

Supplementary Figure 5 RNA conserved motifs of the housekeeping sRNA *ffs*, *ssrA*, *ssrS*, and *RNase P*. RNA motifs were generated on top of Rfam structures with RNA homologs in *E. faecalis* and *faecium* that were retrieved by GlassGO. Motifs were generated with the following number of unique sequences: ffs n=26, SsrA n=55, SsrS n=11 and RNase P n=18.

Supplementary Figure 6 Alignments of conserved sRNAs. Alignments of highly conserved sRNAs, also validated by Northern blot. *E. faecalis* sRNA111/*E. faecium* sRNA_106 correspond to the 23S-methyl RNA motif (Rfam RF01065). sRNA_044 corresponds to a predicted Tbox (RF00230) and sRNA_009 in *E. faecalis* does not correspond to any known motif. Accession numbers for each strain used in the alignment are the same as in the other conservation analyses.

Supplementary Figure 7 Alignments of potentially missed sRNAs. Alignments of sRNAs conserved between *E. faecalis* V583 and *E. faecium* AUS0004. Alignments are listed from top to bottom in order of decreasing percent identity. Genomic context is indicated below each alignment. **(A)** Predicted as an sRNA in *E. faecalis* and *E. faecium.* **(B)** Predicted as an sRNA in *E. faecalis*, but not predicted in *E. faecium.* **(C)** Predicted as an sRNA in *E. faecium*, but not predicted in *E. faecalis.*

Supplementary Table 1 TSS detected in *E. faecalis* V583 and *E. faecium* AUS0004.

Supplementary Table 2 Predicted function of genes associated with conserved −35 promoters.

Supplementary Table 3 5′UTRs and leaderless detected by ANNOgesic for primary and secondary TSS.

Supplementary Table 4 Rfam predictions.

Supplementary Table 5 3′UTRs detected for expressed mRNAs.

Supplementary Table 6 Predicted rho -dependent and independent terminators.

Supplementary Table 7 Predicted sRNAs.

Supplementary Table 8 sRNA conservation.

Supplementary Table 9 mRNA targets prediction.

Supplementary Table 10 Primers used in this study.
