# Supplementary material for: Single-Nucleotide RNA Maps for the Two Major Nosocomial Pathogens Enterococcus faecalis and Enterococcus faecium
Source: Front Cell Infect Microbiol. 2020 Nov 25;10:600325. doi: 10.3389/fcimb.2020.600325 (PMC7724050; doi:10.3389/fcimb.2020.600325)
Supplement: Supplementary Figure 1 — Enterococcus faecalis and E. faecium TSS classifications. Representation of categories for TSS based on expression strength and genomic context assigned by TSSPredator for the chromosome and plasmids in E. faecalis (A) and E. faecium (B): primary (P), secondary (S), internal (I), antisense (A), or orphan (O). [file DataSheet_1.pdf]

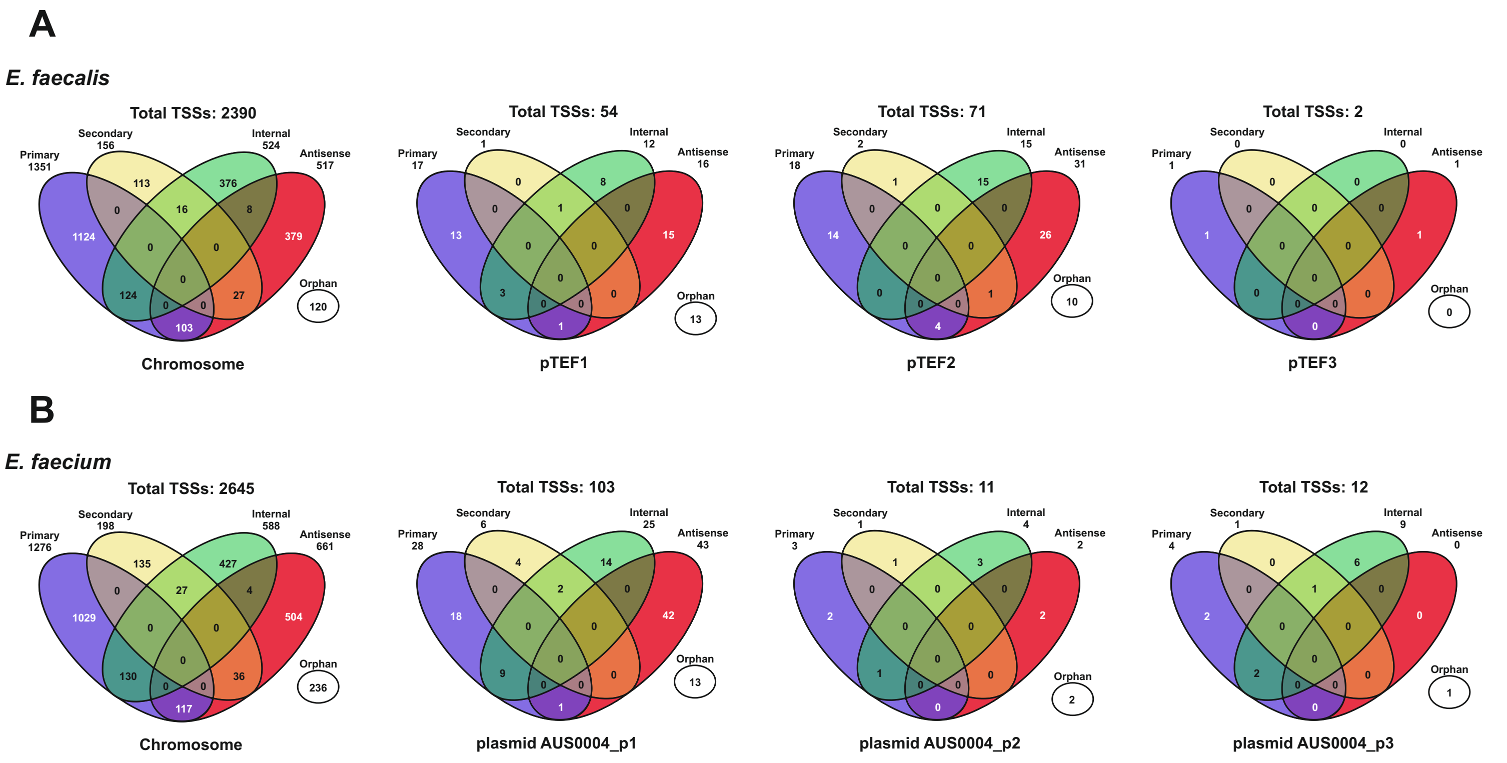

Supplementary Figure 1.

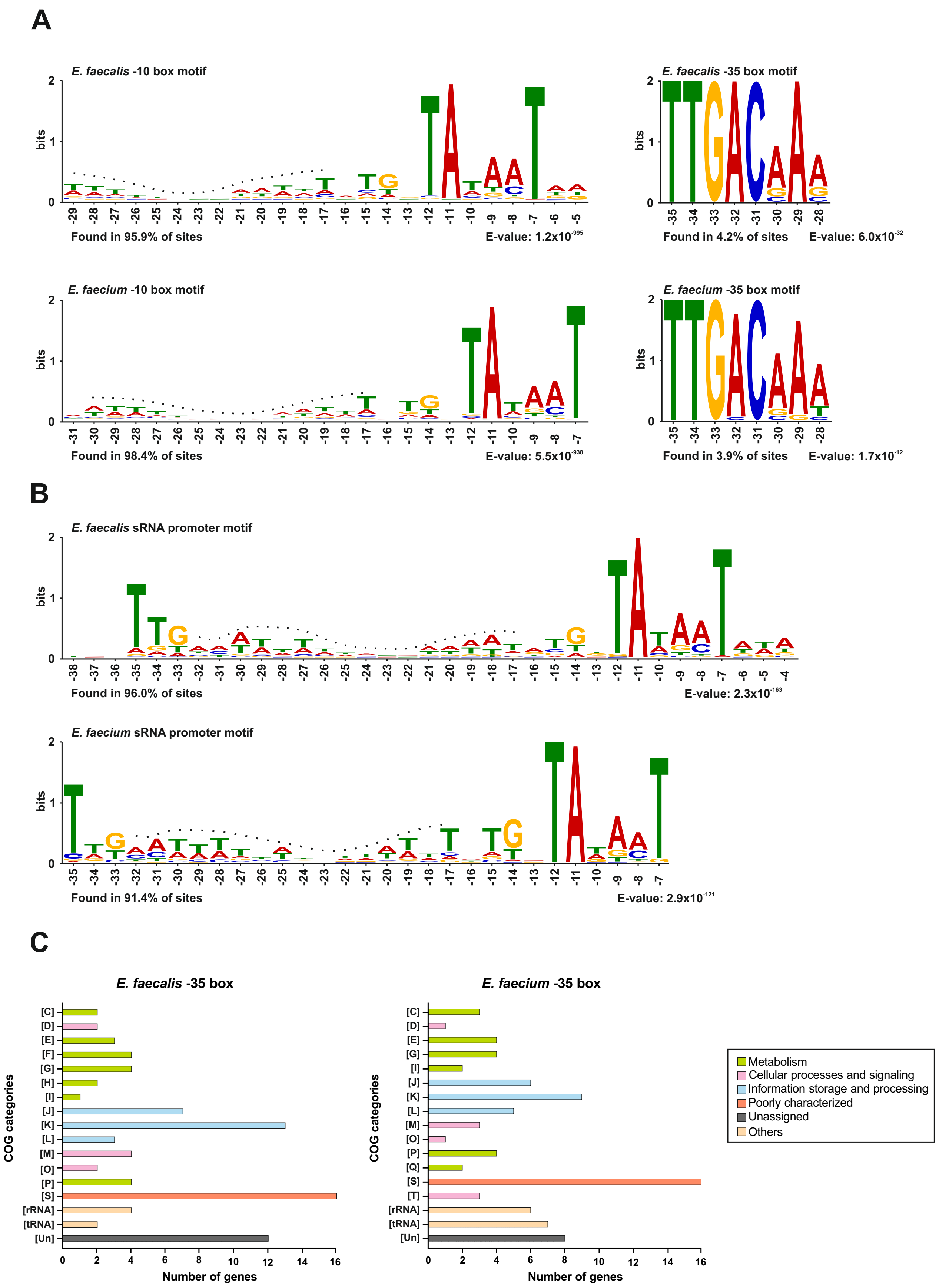

Supplementary Figure 2.

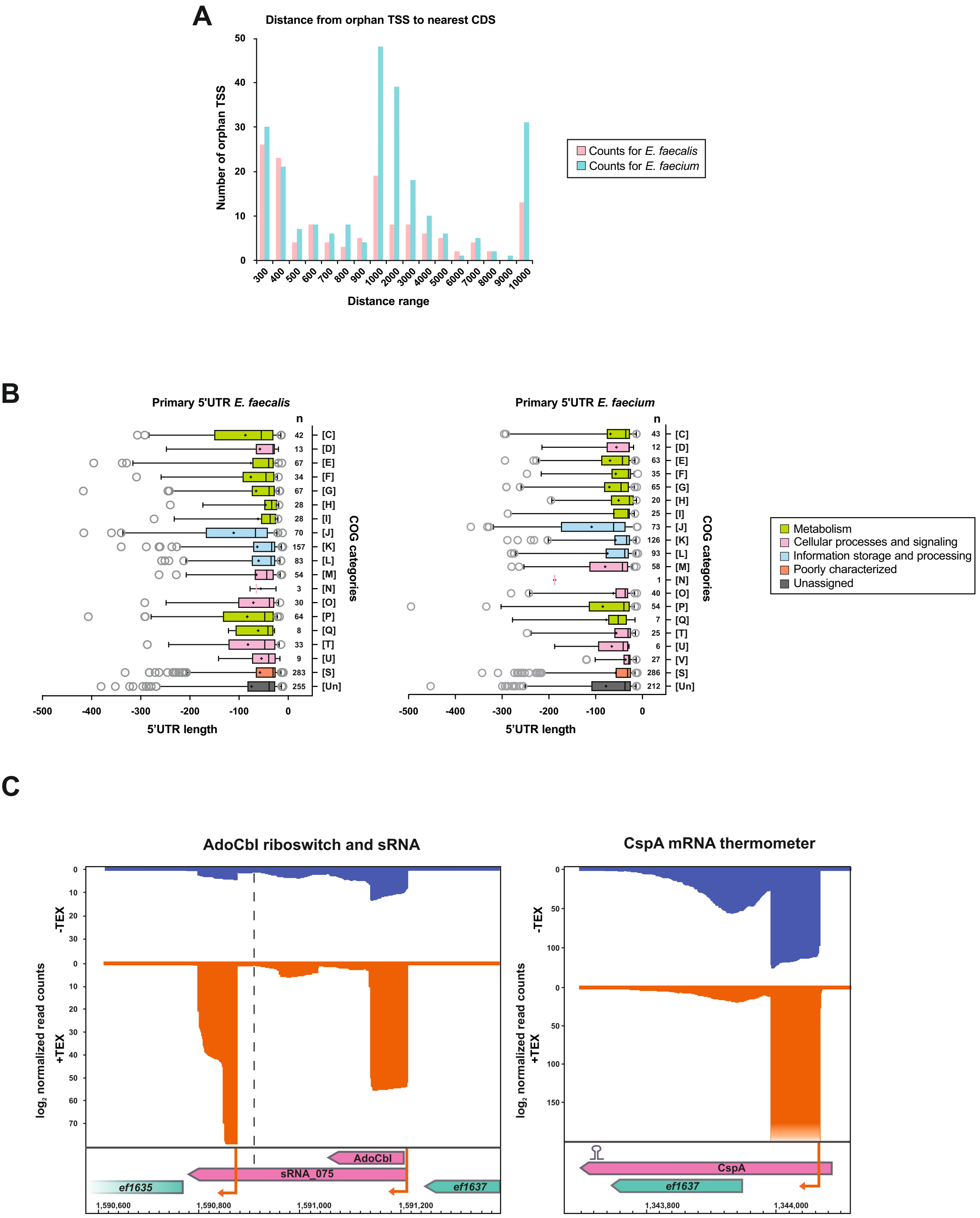

Supplementary Figure 3.

E. faecalis circo plots - plasmids

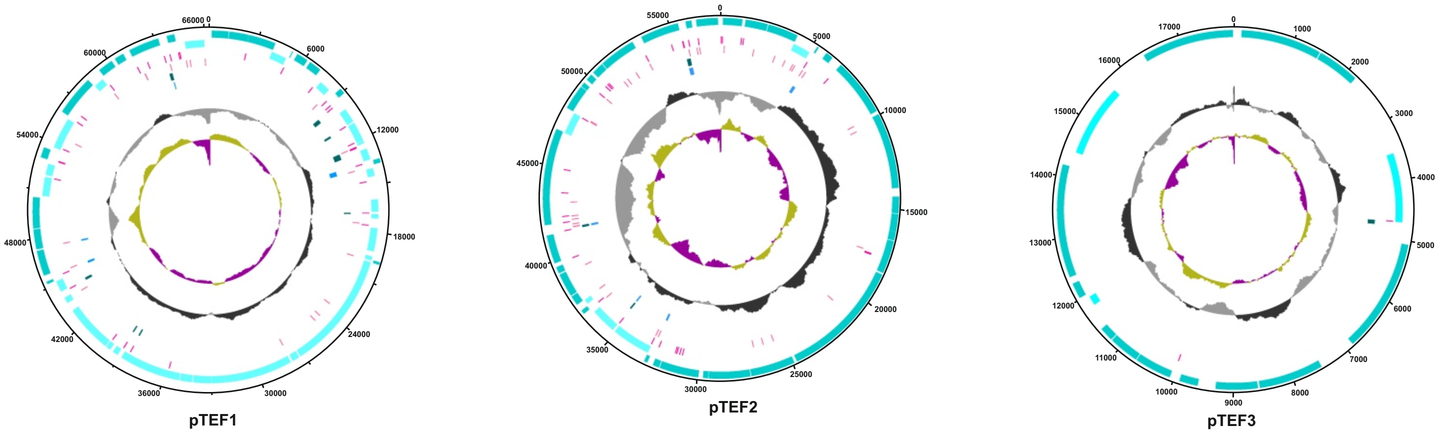

E. faecium circo plots - plasmids

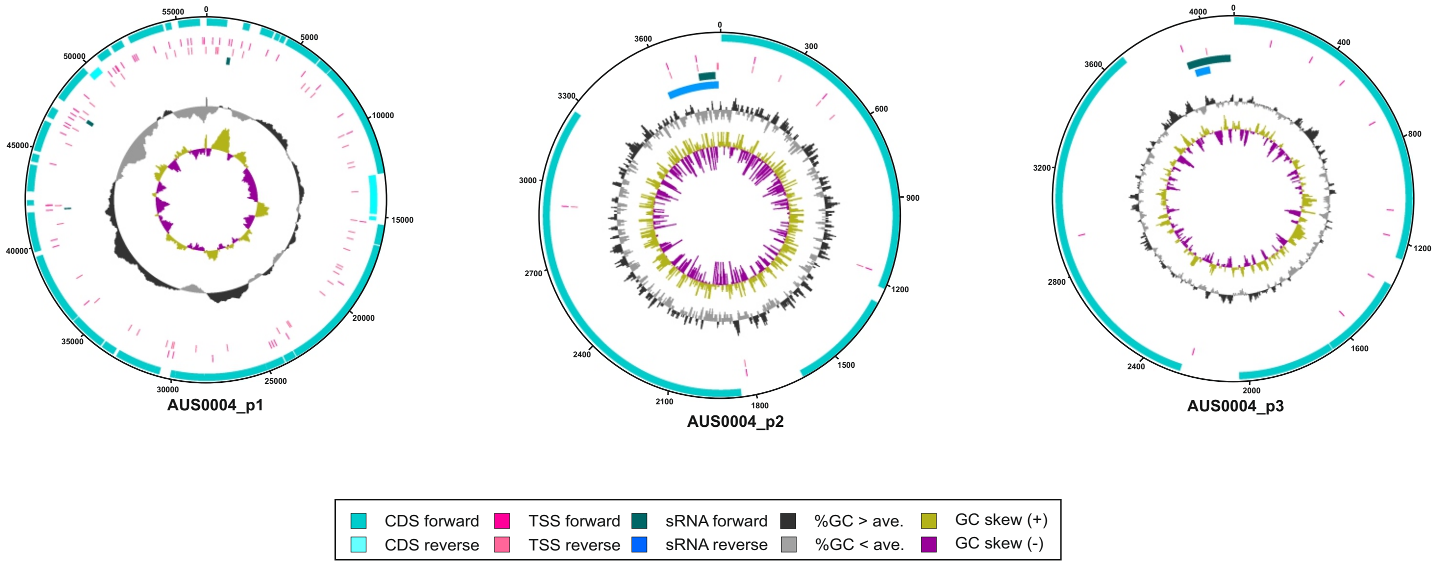

Supplementary Figure 4.

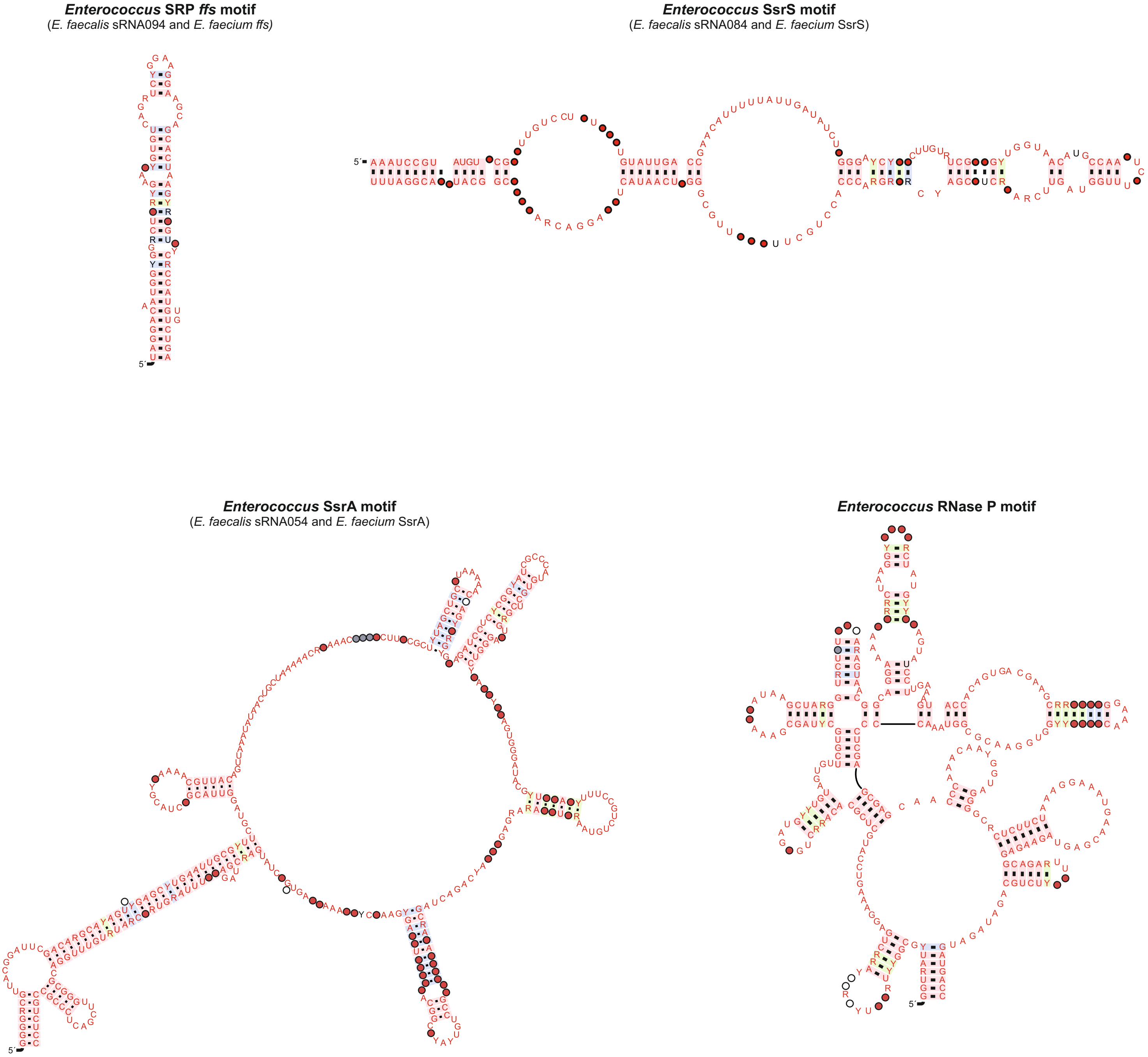

Supplementary Figure 5.

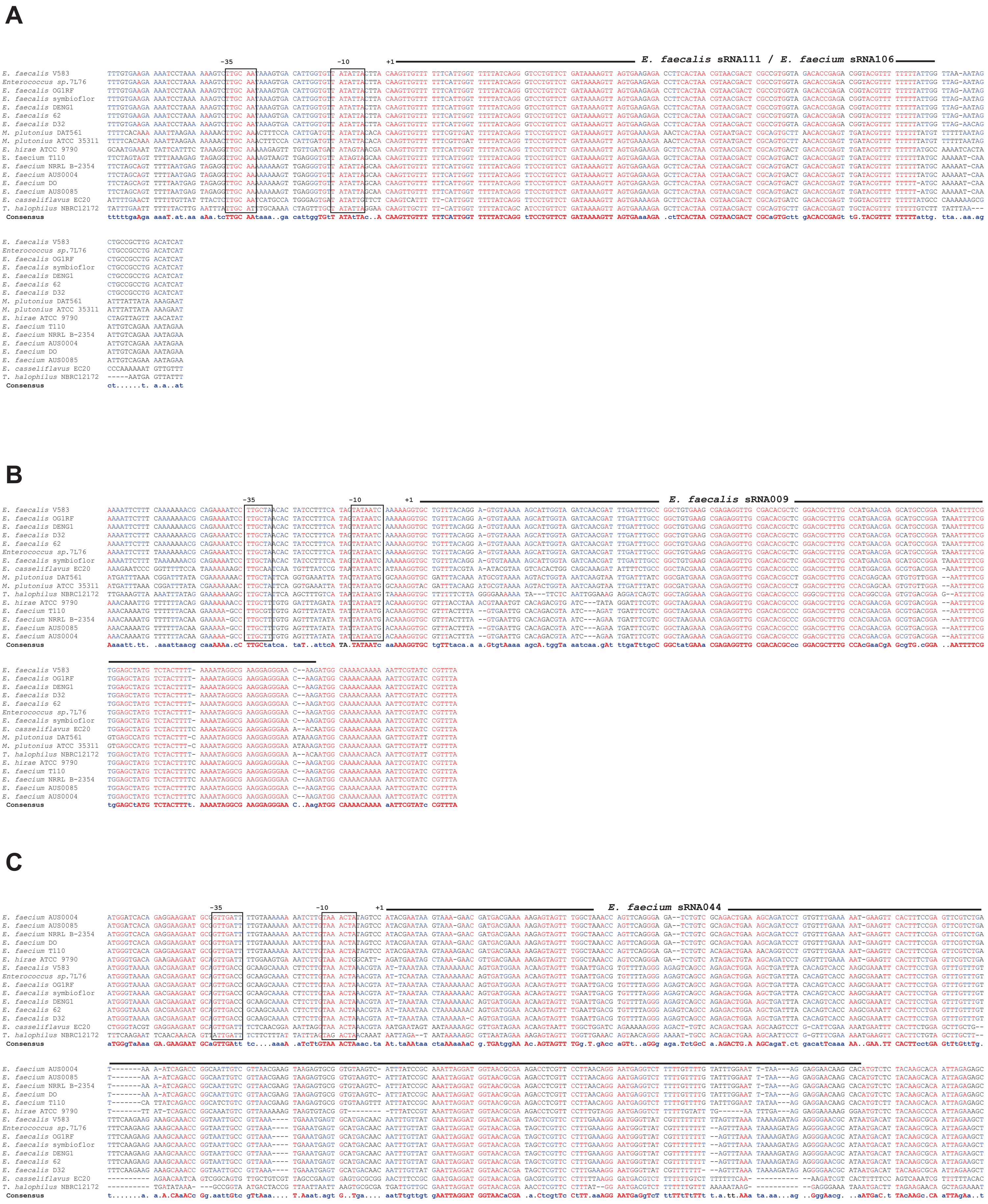

Supplementary Figure 6.

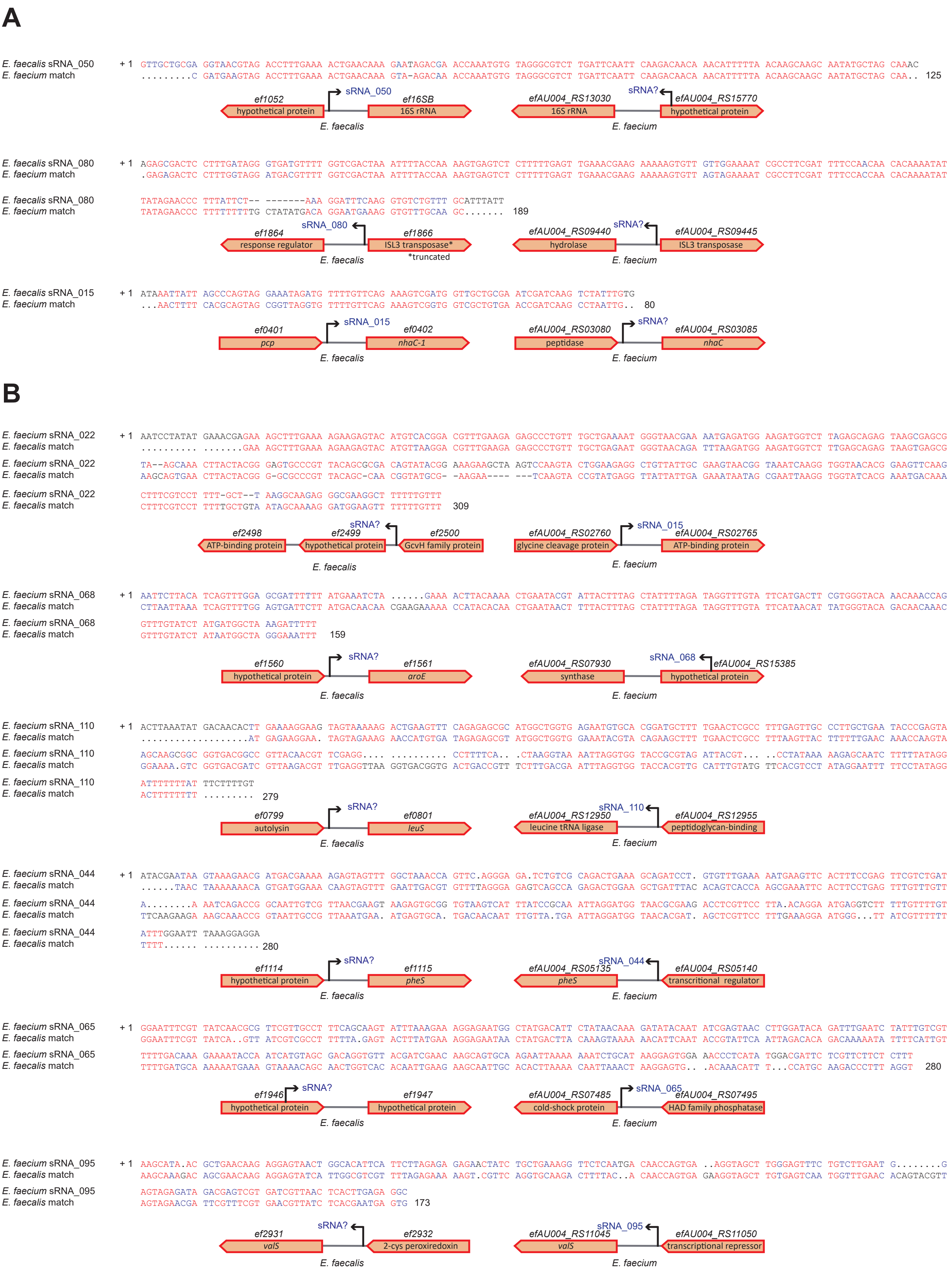

Supplementary Figure 7
