# Supplementary material for: Single-Nucleotide RNA Maps for the Two Major Nosocomial Pathogens Enterococcus faecalis and Enterococcus faecium
Source: Front Cell Infect Microbiol. 2020 Nov 25;10:600325. doi: 10.3389/fcimb.2020.600325 (PMC7724050; doi:10.3389/fcimb.2020.600325)
Supplement: Supplementary Table 10 — Primers used in this study. [file Table_10.docx]

| **Primer name** | **Sequence** | **Predicted sRNA targeted** |
| --- | --- | --- |
| JVO_14699 | ATTCACTTTACAGCTTGCCGAGTGACAG | *ssrA* (*E. faecium*) |
| JVO_14700 | GGTCCCATGGTCGTAGCTTCGAACTAC | *ssrS* (*E. faecium*) |
| JVO_14701 | TCTGCAGTTATACTGCCTTTTTGTCCTGT | *rnpB* (*E. faecium*) |
| JVO_16137 | AGTAGGTCCTCCAAGACACTCTTCCTGTTC | sRNA00029 (*E. faecium*) |
| JVO_16138 | TTTATCACCATACTTGTGAAAGGAGATATT | sRNA00037 (*E. faecium*) |
| JVO_16139 | TTTACCCATTGGCGACTTTCGACGTTTCTG | sRNA00038 (*E. faecium*) |
| JVO_16140 | TCTTTTTCGTCATCGTTCTTTACTTATTCG | sRNA00044 (*E. faecium*) |
| JVO_16141 | CACCCTGCTGATTAGGCAGGGATATAGGAT | sRNA00046 (*E. faecium*) |
| JVO_16142 | AAGGCTTACAATTGGAGTGGTAGTTAATTA | sRNA00059 (*E. faecium*) |
| JVO_16143 | GTAGCCCGGAACGGATTTTATCACCATACT | sRNA00077 (*E. faecium*) |
| JVO_16144 | TTAAAGCGTTCCCCAAAAACCACTTCCCCA | sRNA00078 (*E. faecium*) |
| JVO_16145 | TGAGGAGTAAAAATGAAAAAGTATATAGGG | sRNA00093 (*E. faecium*) |
| JVO_16146 | TGCCAGTTACTCCTCTTGTTCAGCGTTATG | sRNA00095 (*E. faecium*) |
| JVO_16147 | AAAGTAGTGGAAAAAGAGACAAGAAAGAAT | sRNA00097 (*E. faecium*) |
| JVO_16148 | TTTGCCATAGTCAATGCAGTGCATCGACCT | sRNA00098 (*E. faecium*) |
| JVO_16149 | AACCGCCGAGATGGTTAGACTCGACGGAAA | sRNA00101 (*E. faecium*) |
| JVO_16151 | TCGGTGTCAGTCACTGCGAGTCGTTACGT | sRNA00106 (*E. faecium*) |
| JVO_16152 | TCTTACTGTCAACGCTCTATTTTTCATTCT | sRNA00109 (*E. faecium*) |
| JVO_16154 | TCGTGCCTATGTATGGTCTTTTCCCTTGTG | sRNA00119 (*E. faecium*) |
| JVO_16155 | AGGTCGAGAACGTGTCCAGCGTTGCTCGAC | sRNA00121 (*E. faecium*) |
| JVO_16156 | AGGTCGAGAACGTGTCCAGCGTTGCTCGAC | sRNA00122 (*E. faecium*) |
| JVO_16157 | AGAAACGTAAAAGAAAACCACTAGCCCGCC | sRNA00127 (*E. faecium*) |
| JVO_16158 | GGCGGGCTAGTGGTTTTCTTTTACGTTTCT | sRNA00128 (*E. faecium*) |
| JVO_14694 | GTTCGCTTTACAGCGTGCCGAGTGACAG | *ssrA* (*E. faecalis*) |
| JVO_14697 | GGTTCCTCGATCGCAGTATTGAACTAC | *ssrS* (*E. faecalis*) |
| JVO_14698 | CATCTATCTGCAGAGTAAACTCTGCCTC | *rnpB* (*E. faecalis*) |
| JVO_16120 | GCCAGGCAACACTCATGGGA | sRNA_00039 (*E. faecalis*) |
| JVO_16121 | CCCAGCAATGATAACTAGGT | sRNA_00148 (*E. faecalis*) |
| JVO_16122 | ACTCAGTTGGGTAGACTGAG | sRNA_00109 (*E. faecalis*) |
| JVO_16123 | CTCCACGAAAATTTATCCGGCG | sRNA_00009 (*E. faecalis*) |
| JVO_16160 | CTTGCGTGTTATCTGTACG | sRNA_00115 (*E. faecalis*) |
| JVO_16161 | TAGAATAAAGGGTTCTATAA | sRNA_00080 (*E. faecalis*) |
| JVO_16162 | CACGACCAACTTATTTTCCGC | sRNA_00150 (*E. faecalis*) |
| JVO_16117 | GATTTCAAAGTACACAAA | sRNA_00031 (*E. faecalis*) |
| JVO_16118 | TGCCGATGGAAAAGGAGTAT | sRNA_00081 (*E. faecalis*) |
| JVO_16119 | TGTCTACCACGGCGAGTCGT | sRNA_00111 (*E. faecalis*) |
| JVO_16304 | TCGAGGTTTAAATTCATGACAC | sRNA_00021 (*E. faecalis*) |
| JVO_16305 | GCACTTTAGGTGCGTGTTAGCA | sRNA_00027 (*E. faecalis*) |
| JVO_16306 | ATTAGTCGTAAGTGTACAAT | sRNA_00028 (*E. faecalis*) |
| JVO_16307 | CGTTCGTGTAACGTACGAAAC | sRNA_00030 (*E. faecalis*) |
| JVO_16308 | CATTAGGTAGCTTAAAAGAGTG | sRNA_00017 (*E. faecalis*) |
| JVO_16309 | ACAGTCCTTTTACGGTACAATG | sRNA_00019 (*E. faecalis*) |
| JVO_16310 | CAGTAAGTGACCGATTTCAGTC | sRNA_00032 (*E. faecalis*) |
| JVO_16311 | ACATTGACCCTAATCACAACCA | sRNA_00145 (*E. faecalis*) |
| JVO_16312 | GGTTCTTCCTAGTAAGATACAT | sRNA_00121 (*E. faecalis*) |

**Supplementary table 10**: Oligonucleotides used in this study.
